# Supplementary material for: Multi-State Second-Order Nonlinear Optical Switches Incorporating One to Three Benzazolo-Oxazolidine Units: A Quantum Chemistry Investigation
Source: Molecules. 2022 Apr 26;27(9):2770. doi: 10.3390/molecules27092770 (PMC9105456; doi:10.3390/molecules27092770)
Supplement: Supplementary file 1 [file molecules-27-02770-s001.zip › molecules-1681697-supplementary.pdf]

Multi-State Second-Order Nonlinear Optical Switches Incorporating from One to Three Benzazolo-Oxazolidine Units: a Quantum Chemistry Investigation
Supplementary Materials

Pierre Beaujean, Lionel Sanguinet, Vincent Rodriguez, Frédéric Castet, and Benoît Champagne

Version of April 25, 2022

Preamble. The weight, w_i, of a given conformer with a Gibbs free energy ΔG_i^0 (in kJ mol^{-1}) in an ensemble of N conformers $\{\Delta G_j^0 | 0 < i \leq N\}$ and within a Maxwell-Boltzmann (MB) statistic, is computed as:

$$w_i = \frac{e^{\frac{-\Delta G_i^0}{RT}}}{\sum_j^N e^{\frac{-\Delta G_j^0}{RT}}}, \tag{1}$$

where T is the temperature (in K) and R the gas constant (8.3145 J mol^{-1} K^{-1}). The corresponding population, P_i, for conformer i is given by $P_i = 100\, w_i$.

S1 Geometrical parameters

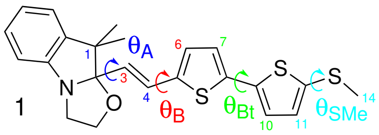

	ΔG^0	P	θ_A	θ_B	θ_{Bt}	θ_{SMe}	BLA$_{2,5}$
				**C**			
1	0.0	49.2	112.5	-179.9	-151.2	-95.2	0.147
2	2.0	21.7	112.8 (+0.3)	16.1 (-164.0)	150.7 (-58.2)	95.8 (-169.0)	0.150
3	3.0	14.7	112.6 (+0.1)	-177.2 (+2.6)	-31.0 (+120.2)	-94.1 (+1.2)	0.147
4	5.4	5.5	112.1 (-0.4)	10.4 (-169.7)	31.3 (-177.6)	-95.5 (-0.3)	0.150
5	5.9	4.5	112.0 (-0.5)	179.4 (-0.7)	-152.1 (-0.9)	-1.4 (+93.9)	0.146
6	7.8	2.1	112.1 (-0.5)	-177.9 (+2.0)	-32.4 (+118.7)	-0.6 (+94.6)	0.147
7	9.4	1.1	112.2 (-0.3)	11.4 (-168.7)	33.2 (-175.6)	-1.0 (+94.2)	0.149
8	9.4	1.1	112.1 (-0.4)	15.9 (-164.2)	150.4 (-58.5)	0.7 (+95.9)	0.149
				**O**			
1	0.0	54.6	2.5	-179.1	156.6	-95.5	0.062
2	2.7	18.7	3.2 (+0.7)	-178.9 (+0.1)	-24.5 (+179.0)	-95.2 (+0.2)	0.062
3	3.2	15.1	2.4 (-0.1)	178.8 (-2.1)	-162.6 (+40.8)	-3.0 (+92.5)	0.056
4	6.3	4.2	2.7 (+0.2)	-1.6 (+177.5)	-154.6 (+48.8)	-93.5 (+2.0)	0.065
5	7.6	2.5	-162.2 (-164.7)	-177.0 (+2.1)	156.6 (+0.0)	-94.7 (+0.7)	0.062
6	8.4	1.9	1.8 (-0.7)	-1.8 (+177.3)	25.5 (-131.1)	95.3 (-169.2)	0.064
7	8.8	1.5	1.9 (-0.6)	0.2 (+179.3)	159.2 (+2.7)	1.1 (+96.6)	0.059
8	10.0	1.0	-161.2 (-163.7)	-178.9 (+0.1)	25.3 (-131.2)	96.5 (-168.0)	0.063
9	12.0	0.4	-163.1 (-165.6)	-176.8 (+2.3)	162.7 (+6.1)	3.2 (+98.6)	0.056

Table S1: Thermochemical and geometrical features of the different conformers of compound **1**, as evaluated at the ωB97X-D/6-311G(d)/IEF-PCM(acetonitrile) level of theory: relative Gibbs free energy (ΔG^0, kJ mol^{-1}), corresponding MB population (P, %) at 298.15 K, torsional angles (θ, °, with the relative difference with respect to the most stable conformer in parentheses) and bond length alternation (BLA, Å).

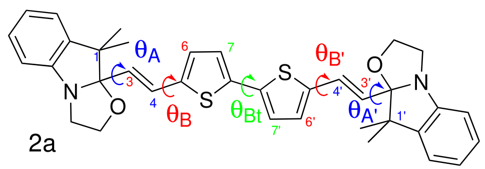

2a

	ΔG^0	P	θ_A	θ_B	θ_{Bt}	$\theta_{B'}$	$\theta_{A'}$	$BLA_{2,5}$	$BLA_{2',5'}$
					**CC**				
1	0.0	45.5	-108.7	178.6	-154.4	177.1	-112.4	0.147	0.147
2	9.1	1.2	-108.7 (+0.0)	-15.2 (+166.2)	-153.6 (+0.8)	178.9 (+1.8)	108.5 (-139.1)	0.149	0.147
3	0.8	32.5	112.1 (-139.1)	179.6 (+1.0)	-155.0 (-0.6)	178.0 (+0.9)	108.5 (-139.1)	0.147	0.147
4	3.3	12.0	-109.0 (-0.3)	177.7 (-0.9)	-154.6 (-0.2)	178.5 (+1.4)	108.6 (-139.0)	0.147	0.147
5	11.9	0.4	-108.5 (+0.2)	-3.1 (+178.3)	-152.0 (+2.4)	8.0 (-169.1)	108.6 (-139.0)	0.149	0.149
6	7.7	2.1	112.0 (-139.3)	8.7 (-169.9)	-153.3 (+1.1)	-0.2 (-177.3)	108.9 (-138.7)	0.149	0.149
7	5.4	5.1	-108.9 (-0.1)	178.3 (-0.3)	-153.2 (+1.2)	-13.3 (+169.6)	-112.1 (+0.3)	0.147	0.149
8	8.8	1.3	-108.5 (+0.2)	178.6 (+0.0)	-153.9 (+0.5)	11.3 (-165.8)	108.8 (-138.8)	0.147	0.149
					**CO**				
1	0.0	53.1	-1.8	-179.6	-158.8	179	-111.7	0.060	0.147
2	3.4	13.3	-2.4 (-0.6)	179.6 (-0.9)	-161.6 (-2.8)	179.5 (+0.5)	108.9 (-139.4)	0.060	0.147
3	4.2	9.8	-2.9 (-1.0)	178.5 (-1.9)	-161.6 (-2.7)	-12.9 (+168.1)	-112.3 (-0.7)	0.059	0.149
4	5.1	6.8	-2.1 (-0.3)	-1.8 (+177.8)	-158.2 (+0.6)	177.7 (-1.2)	-112.3 (-0.7)	0.063	0.147
5	5.6	5.6	-2.9 (-1.0)	179.3 (-1.2)	-161.9 (-3.1)	4.9 (-174.0)	108.7 (-139.6)	0.059	0.149
6	6.9	3.2	-2.5 (-0.7)	-0.9 (+178.7)	-160.2 (-1.4)	-10.0 (+171.1)	-111.9 (-0.3)	0.061	0.149
7	7.1	3.0	-2.7 (-0.9)	-2.0 (+177.6)	-158.5 (+0.3)	179.4 (+0.4)	109.0 (-139.3)	0.063	0.147
8	8.0	2.1	-3.3 (-1.5)	0.9 (-179.6)	158.0 (-43.1)	12.1 (-166.9)	108.8 (-139.6)	0.062	0.150
9	9.2	1.3	157.1 (+158.9)	177.1 (-3.4)	-156.4 (+2.5)	178.7 (-0.3)	-112.1 (-0.4)	0.066	0.147
10	9.8	1.0	157.0 (+158.8)	175.7 (-4.7)	-160.3 (-1.5)	179.4 (+0.4)	109.1 (-139.2)	0.066	0.147
11	10.7	0.7	156.9 (+158.7)	176.5 (-3.9)	-159.7 (-0.9)	-11.3 (+169.7)	-111.5 (+0.2)	0.065	0.149
					**OO**				
1	0.0	64.8	-3.7	179.9	154.7	-179.5	3.9	0.070	0.070
2	2.9	20.0	-2.5 (+1.2)	1.8 (-178.1)	158.2 (+3.5)	1.7 (-178.8)	2.7 (-1.1)	0.072	0.072
3	3.6	15.2	-3.0 (+0.7)	179.7 (-0.3)	178.1 (+23.3)	1.1 (-179.4)	2.7 (-1.1)	0.069	0.072

Table S2: Thermochemical and geometrical features of the different conformers of compound **2a**, as evaluated at the ωB97X-D/6-311G(d)/IEF-PCM(acetonitrile) level of theory: relative Gibbs free energy (ΔG^0, kJ mol^{-1}), corresponding MB population (P, %) at 298.15 K, torsional angles (θ, °, with the relative difference with respect to the most stable conformer in parentheses) and bond length alternation (BLA, Å).

2b

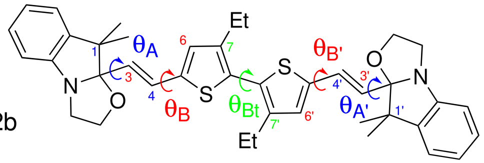

	ΔG^0	P	θ_A	θ_B	θ_{Bt}	$\theta_{B'}$	$\theta_{A'}$	$\text{BLA}_{2,5}$	$\text{BLA}_{2',5'}$
				**CC**					
1	0.0	71.5	-109.2	179	90.9	178.5	-112.5	0.148	0.148
2	4.8	10.2	111.9 (-138.9)	-178.4 (+2.6)	91.7 (+0.9)	-178.6 (+2.9)	109.3 (-138.2)	0.148	0.148
3	5.7	7.3	-109.3 (-0.1)	7.8 (-171.2)	92.4 (+1.5)	-179.0 (+2.5)	108.9 (-138.6)	0.150	0.148
4	6.3	5.6	-108.8 (+0.4)	179.8 (+0.8)	91.6 (+0.8)	-179.9 (+1.6)	108.6 (-138.8)	0.148	0.148
5	8.0	2.8	-109.1 (+0.1)	179.1 (+0.1)	89.4 (-1.5)	-12.4 (+169.1)	-111.3 (+1.2)	0.148	0.150
6	9.5	1.6	-108.9 (+0.3)	179.7 (+0.7)	91.5 (+0.6)	6.4 (-172.0)	108.6 (-138.9)	0.148	0.150
7	11.0	0.8	112.3 (-138.5)	14.3 (-164.7)	92.5 (+1.7)	10.2 (-168.3)	108.3 (-139.2)	0.150	0.150
				**CO**					
1	0.0	90.4	-1.2	-178.2	96.4	-179.9	-112	0.068	0.148
2	7.0	5.3	-2.0 (-0.7)	-178.9 (-0.8)	95.6 (-0.8)	-179.2 (+0.7)	108.9 (-139.0)	0.068	0.149
3	9.5	1.9	-1.7 (-0.5)	-179.2 (-1.1)	93.9 (-2.5)	-14.7 (+165.3)	-112.3 (-0.2)	0.067	0.151
4	10.0	1.6	-3.0 (-1.8)	-0.3 (+177.8)	93.1 (-3.3)	178.3 (-1.7)	-113.2 (-1.2)	0.070	0.148
5	12.3	0.6	-2.3 (-1.1)	179.6 (-2.3)	94.1 (-2.3)	11.3 (-168.8)	108.6 (-139.4)	0.067	0.151
				**OO**					
1	0.0	64.9	-1.9	-178.7	95.3	-179.1	2.9	0.072	0.072
2	1.7	32.6	-3.4 (-1.5)	179.7 (-1.6)	93.1 (-2.2)	-0.5 (+178.6)	2.2 (-0.7)	0.072	0.074
3	9.4	1.5	-2.9 (-1.0)	0.0 (+178.7)	96.3 (+1.0)	4.7 (-176.2)	4.7 (+1.8)	0.073	0.074
4	10.2	1.0	-4.4 (-2.5)	178.9 (-2.4)	94.4 (-0.9)	9.0 (-171.9)	-151.2 (-154.1)	0.071	0.082

Table S3: Thermochemical and geometrical features of the different conformers of compound **2b**, as evaluated at the ωB97X-D/6-311G(d)/IEF-PCM(acetonitrile) level of theory: relative Gibbs free energy (ΔG^0, kJ mol^{-1}), corresponding MB population (P, %) at 298.15 K, torsional angles (θ, °, with the relative difference with respect to the most stable conformer in parentheses) and bond length alternation (BLA, Å).

2c

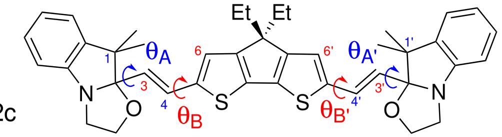

	ΔG^0	P	θ_A	θ_B	$\theta_{B'}$	$\theta_{A'}$	$BLA_{2,5}$	$BLA_{2',5'}$
**CC**								
1	0.0	41.4	-108.5	179.3	178.7	-111.8	0.145	0.145
2	1.3	24.1	111.6 (-139.9)	179.5 (+0.2)	179.4 (+0.7)	108.5 (-139.6)	0.145	0.145
3	1.9	19.1	-108.3 (+0.2)	179.5 (+0.2)	179.6 (+0.9)	108.5 (-139.7)	0.145	0.145
4	4.5	6.6	-108.7 (-0.2)	178.2 (-1.1)	-2.2 (+179.1)	-112.4 (-0.6)	0.145	0.147
5	5.3	4.9	-109.2 (-0.7)	-0.3 (-179.6)	-179.5 (+1.8)	108.9 (-139.3)	0.147	0.145
6	7.8	1.8	-108.8 (-0.3)	178.0 (-1.3)	12.2 (-166.5)	108.2 (-140.0)	0.145	0.147
7	8.5	1.4	112.0 (-139.5)	12.7 (-166.6)	9.4 (-169.3)	108.1 (-140.1)	0.147	0.147
8	9.9	0.8	-109.0 (-0.5)	-2.5 (+178.2)	5.8 (-172.9)	108.0 (-140.2)	0.147	0.147
**CO**								
1	0.0	59.9	1.9	179.5	-179.9	108.9	0.042	0.146
2	2.9	18.5	2.4 (+0.5)	-179.9 (+0.7)	177.4 (-2.7)	-112.6 (+138.5)	0.042	0.146
3	4.9	8.3	2.8 (+0.9)	-179.6 (+1.0)	-5.6 (+174.3)	-112.8 (+138.3)	0.041	0.148
4	6.5	4.3	1.6 (-0.3)	-1.0 (+179.6)	177.2 (-2.9)	-112.7 (+138.4)	0.044	0.146
5	7.7	2.7	2.1 (+0.2)	179.6 (+0.2)	14.3 (-165.8)	108.8 (-0.1)	0.041	0.148
6	8.2	2.2	2.3 (+0.4)	1.0 (-178.5)	-0.9 (+179.0)	-112.6 (+138.5)	0.042	0.148
7	8.9	1.6	1.7 (-0.2)	0.1 (-179.3)	14.7 (-165.4)	108.6 (-0.3)	0.042	0.148
8	9.6	1.3	2.3 (+0.4)	0.5 (-178.9)	179.9 (-0.2)	108.9 (-0.1)	0.044	0.146
9	10.7	0.8	-165.4 (-167.3)	-177.8 (+2.7)	177.3 (-2.8)	-112.6 (+138.5)	0.042	0.146
10	12.4	0.4	-164.8 (-166.7)	-178.0 (+2.5)	4.1 (-176.0)	-112.4 (+138.7)	0.041	0.148
**OO**								
1	0.0	66.1	-3.6	177.4	179	1.2	0.063	0.062
2	2.8	21.1	-1.5 (+2.2)	2.4 (-175.0)	1.9 (-177.1)	3.0 (+1.8)	0.064	0.063
3	4.1	12.8	-1.4 (+2.2)	-178.2 (+4.4)	0.2 (-178.8)	2.5 (+1.3)	0.062	0.064

Table S4: Thermochemical and geometrical features of the different conformers of compound **2c**, as evaluated at the ωB97X-D/6-311G(d)/IEF-PCM(acetonitrile) level of theory: relative Gibbs free energy (ΔG^0, kJ mol^{-1}), corresponding MB population (P, %) at 298.15 K, torsional angles (θ, °, with the relative difference with respect to the most stable conformer in parentheses) and bond length alternation (BLA, Å).

2d

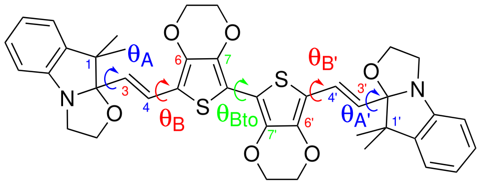

	ΔG^0	P	θ_A	θ_B	θ_{Bto}	$\theta_{B'}$	$\theta_{A'}$	$BLA_{2,5}$	$BLA_{2',5'}$
					**CC**				
1	0.0	82.1	61.2	-179	179.1	180	109.3	0.142	0.142
2	3.8	17.9	73.2 (+12.1)	175.7 (-5.3)	178.5 (-0.6)	177.7 (-2.2)	108.5 (-0.8)	0.142	0.142
					**CO**				
1	0.0	66.3	-5	-178.9	-179.9	177.4	-111.1	0.036	0.144
2	3.7	14.7	-5.1 (-0.1)	-179.7 (-0.8)	179.4 (-0.7)	0.9 (-176.5)	-112.8 (-1.8)	0.036	0.142
3	3.8	14.1	-7.5 (-2.5)	1.2 (-179.9)	178.6 (-1.5)	174.9 (-2.5)	-112.9 (-1.9)	0.040	0.144
4	8.2	2.5	-5.8 (-0.8)	-179.5 (-0.7)	179.9 (-0.2)	178.1 (+0.7)	109.0 (-140.0)	0.037	0.144
5	8.2	2.4	-6.1 (-1.1)	-179.7 (-0.8)	179.4 (-0.7)	8.8 (-168.6)	109.4 (-139.6)	0.035	0.143
					**OO**				
1	0.0	82.9	-30.7	179.7	179.5	-179	7.2	0.055	0.055
2	5.4	9.4	-31.3 (-0.6)	3.0 (-176.7)	179.6 (+0.1)	2.4 (-178.6)	6.4 (-0.7)	0.056	0.055
3	6.1	7.0	-29.1 (+1.6)	-178.8 (+1.5)	-179.7 (+0.8)	2.9 (-178.1)	7.2 (+0.0)	0.053	0.057
4	11.8	0.7	128.0 (+158.7)	176.0 (-3.7)	179.5 (+0.0)	-179.7 (-0.7)	6.5 (-0.6)	0.059	0.053

Table S5: Thermochemical and geometrical features of the different conformers of compound **2d**, as evaluated at the ωB97X-D/6-311G(d)/IEF-PCM(acetonitrile) level of theory: relative Gibbs free energy (ΔG^0, kJ mol^{-1}), corresponding MB population (P, %) at 298.15 K, torsional angles (θ, °, with the relative difference with respect to the most stable conformer in parentheses) and bond length alternation (BLA, Å).

2e

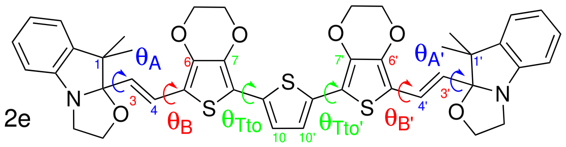

	ΔG^0	P	θ_A	θ_B	θ_{Tto}	$\theta_{Tto'}$	$\theta_{B'}$	$\theta_{A'}$	$BLA_{2,5}$	$BLA_{2',5'}$
					**CC**					
1	0.0	19.3	112.5	-176.8	-177.8	-179.3	0.2	-112.9	0.142	0.142
2	0.9	13.3	112.0 (-0.5)	-177.7 (-0.9)	-178.6 (-0.8)	15.9 (-164.8)	176.5 (+176.3)	-112.3 (+0.7)	0.142	0.142
3	1.0	12.8	112.3 (-0.2)	-176.9 (-0.1)	179.6 (-2.6)	178.9 (-1.8)	178.7 (+178.5)	108.7 (-138.3)	0.142	0.142
4	1.6	10.0	113.0 (+0.5)	-0.3 (+176.5)	-178.2 (-0.4)	178.2 (-2.5)	0.3 (+0.0)	-113.0 (-0.1)	0.142	0.142
5	1.8	9.2	111.9 (-0.5)	-177.1 (-0.3)	178.9 (-3.3)	177.8 (-2.9)	7.8 (+7.5)	109.0 (-138.0)	0.142	0.142
6	1.9	8.9	112.1 (-0.4)	-177.1 (-0.3)	-16.8 (+161.0)	16.8 (-163.9)	177.1 (+176.9)	-112.1 (+0.8)	0.142	0.142
7	2.0	8.7	111.9 (-0.5)	-176.5 (+0.2)	179.1 (-3.1)	16.4 (-164.3)	-1.0 (-1.2)	-113.6 (-0.7)	0.142	0.142
8	2.6	6.8	111.9 (-0.6)	-177.3 (-0.5)	179.8 (-2.5)	15.5 (-165.2)	177.7 (+177.5)	108.3 (-138.8)	0.142	0.143
9	3.0	5.6	113.5 (+1.0)	0.7 (+177.4)	-174.8 (+2.9)	19.2 (-161.5)	179.8 (+179.6)	109.1 (-138.0)	0.142	0.143
10	5.0	2.5	-108.6 (+138.9)	-177.6 (-0.8)	-18.2 (+159.6)	18.1 (-162.6)	177.7 (+177.4)	108.6 (-138.5)	0.143	0.143
11	6.0	1.7	-109.6 (+137.9)	-10.5 (+166.2)	-178.3 (-0.5)	15.1 (-165.6)	175.4 (+175.2)	-112.6 (+0.4)	0.142	0.142
12	6.9	1.2	112.1 (-0.4)	-177.1 (-0.3)	-179.3 (-1.5)	18.4 (-162.3)	7.7 (+7.4)	109.0 (-138.0)	0.142	0.142
					**CO**					
1	0.0	45.8	-111.9	177.3	-177.9	179.3	-179.3	6.8	0.143	0.042
2	3.4	11.7	-113.2 (-1.3)	-1.0 (-178.3)	178.3 (-3.8)	178.2 (-1.1)	-179.7 (-0.4)	5.4 (-1.4)	0.142	0.041
3	3.6	10.8	-112.2 (-0.3)	176.4 (-0.9)	14.1 (-168.1)	179.5 (+0.2)	-179.7 (-0.4)	5.3 (-1.5)	0.143	0.043
4	3.8	10.0	108.2 (-139.9)	177.6 (+0.4)	178.4 (-3.7)	177.1 (-2.2)	-178.4 (+0.9)	6.6 (-0.2)	0.143	0.042
5	4.2	8.5	108.9 (-139.2)	179.0 (+1.7)	-179.1 (-1.2)	10.8 (-168.5)	-179.1 (+0.2)	6.4 (-0.4)	0.143	0.042
6	6.2	3.7	109.1 (-139.0)	8.7 (-168.5)	-179.4 (-1.6)	178.2 (-1.1)	-179.9 (-0.6)	6.5 (-0.3)	0.142	0.041
7	7.2	2.5	-113.0 (-1.2)	0.1 (-177.1)	179.1 (-3.0)	7.2 (-172.1)	-179.5 (-0.2)	6.7 (-0.1)	0.142	0.041
8	7.6	2.1	-111.7 (+0.1)	176.9 (-0.4)	11.7 (-170.4)	6.5 (-172.8)	-179.8 (-0.4)	6.2 (-0.6)	0.143	0.042
9	7.8	1.9	-113.9 (-2.0)	-0.8 (-178.0)	14.5 (-167.6)	178.5 (-0.8)	-179.2 (+0.1)	6.3 (-0.5)	0.142	0.042
10	8.4	1.5	108.8 (-139.4)	177.9 (+0.7)	8.3 (-173.8)	175.0 (-4.3)	-179.8 (-0.5)	6.2 (-0.6)	0.143	0.043
11	8.6	1.4	109.0 (-139.1)	8.2 (-169.0)	14.0 (-168.2)	178.7 (-0.6)	-179.4 (-0.0)	6.0 (-0.8)	0.142	0.042
					**OO**					
1	0.0	80.9	-6.3	179.2	179.3	176.5	179.1	-53.4	0.046	0.046
2	4.0	16.4	-6.2 (+0.1)	179.7 (+0.5)	-178.5 (+2.1)	13.1 (-163.3)	-179.6 (+1.2)	-52.8 (+0.6)	0.047	0.046
3	8.4	2.7	-6.6 (-0.2)	179.2 (-0.0)	179.6 (+0.2)	10.9 (-165.5)	-176.1 (+4.8)	152.5 (-154.1)	0.046	0.051

Table S6: Thermochemical and geometrical features of the different conformers of compound **2e**, as evaluated at the ωB97X-D/6-311G(d)/IEF-PCM(acetonitrile) level of theory: relative Gibbs free energy (ΔG^0, kJ mol^{-1}), corresponding MB population (P, %) at 298.15 K, torsional angles (θ, °, with the relative difference with respect to the most stable conformer in parentheses) and bond length alternation (BLA, Å).

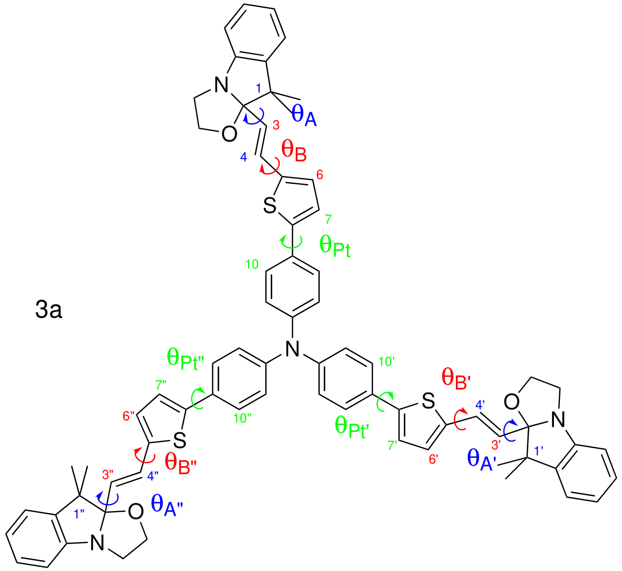

3a

	ΔG^0	P	θ_A	θ_B	θ_{Pt}	$\theta_{A'}$	$\theta_{B'}$	$\theta_{Pt'}$	$\theta_{A"}$	$\theta_{B"}$	$\theta_{Pt"}$
**CCC**											
1	0.0	32.7	111.3	178.6	29.8	112.3	179.8	31.2	112	-178.5	148
2	1.3	19.1	112.5 (+1.1)	-177.4 (+4.0)	149.0 (+119.2)	112.4 (+0.1)	-177.5 (+2.7)	148.9 (+117.6)	112.3 (+0.3)	-177.8 (+0.7)	-30.9 (-179.0)
3	2.6	11.5	112.2 (+0.9)	-177.8 (+3.6)	148.5 (+118.7)	-109.0 (+138.7)	-179.7 (+0.5)	148.2 (+117.0)	-108.8 (+139.2)	-178.9 (-0.4)	-30.5 (-178.5)
4	2.7	11.0	112.2 (+0.9)	-177.6 (+3.8)	149.1 (+119.3)	-109.1 (+138.6)	-179.4 (+0.8)	148.2 (+116.9)	112.0 (+0.0)	-178.2 (+0.3)	-31.1 (-179.1)
5	4.6	5.0	111.8 (+0.5)	-178.1 (+3.3)	148.6 (+118.8)	-108.9 (+138.9)	-179.4 (+0.8)	148.7 (+117.5)	-109.1 (+138.8)	8.9 (-172.5)	-29.5 (-177.5)
6	5.4	3.8	111.8 (+0.5)	179.3 (+0.7)	30.1 (+0.4)	-109.3 (+138.4)	177.3 (-2.5)	29.9 (-1.3)	-109.1 (+138.9)	-179.5 (-0.9)	148.7 (+0.7)
7	5.5	3.5	-108.7 (+140.0)	-179.0 (+2.4)	148.7 (+118.9)	-109.1 (+138.6)	179.7 (-0.1)	148.5 (+117.2)	-108.9 (+139.1)	-179.4 (-0.9)	-31.4 (-179.4)
8	6.4	2.5	112.7 (+1.4)	-179.1 (+2.3)	31.9 (+2.1)	-108.7 (+139.0)	177.8 (-2.0)	30.6 (-0.6)	111.9 (-0.1)	-177.9 (+0.6)	148.5 (+0.5)
9	6.4	2.4	112.2 (+0.9)	12.6 (-166.1)	148.1 (+118.4)	-108.4 (+139.3)	-178.1 (+2.1)	149.7 (+118.5)	-108.7 (+139.3)	-179.3 (-0.8)	-30.8 (-178.8)
10	6.6	2.3	112.2 (+0.9)	12.5 (-166.1)	31.4 (+1.7)	112.9 (+0.6)	-179.6 (+0.6)	31.4 (+0.2)	112.6 (+0.6)	-177.0 (+1.5)	149.1 (+1.1)
11	7.2	1.8	112.8 (+1.5)	-179.0 (+2.4)	31.4 (+1.6)	-108.6 (+139.1)	178.3 (-1.5)	30.4 (-0.9)	-109.3 (+138.7)	4.0 (-177.4)	148.9 (+0.8)
12	7.4	1.7	-108.7 (+140.0)	178.0 (-0.6)	30.8 (+1.1)	-108.6 (+139.1)	179.1 (-0.7)	31.8 (+0.6)	-109.2 (+138.7)	179.7 (-1.8)	147.8 (-0.3)
13	10.4	0.5	158.3 (+47.0)	-100.1 (+81.3)	30.9 (+1.2)	-108.7 (+139.0)	-12.1 (+168.1)	30.8 (-0.5)	-114.9 (+133.1)	-78.7 (+99.8)	148.2 (+0.2)
14	10.5	0.5	112.3 (+0.9)	3.3 (-175.3)	30.8 (+1.0)	-108.6 (+139.2)	178.3 (-1.5)	30.9 (-0.3)	112.5 (+0.4)	-177.3 (+1.2)	148.7 (+0.7)
15	10.6	0.5	111.8 (+0.5)	4.6 (-174.1)	30.9 (+1.1)	-109.0 (+138.7)	177.7 (-2.1)	30.4 (-0.8)	-108.7 (+139.3)	-179.1 (-0.6)	148.6 (+0.5)
16	10.6	0.5	112.0 (+0.7)	9.6 (-169.0)	30.4 (+0.6)	-108.4 (+139.3)	-12.0 (+168.2)	30.8 (-0.4)	112.9 (+0.9)	-179.2 (-0.7)	-148.2 (+63.8)
17	11.0	0.4	111.7 (+0.4)	11.4 (-167.2)	31.0 (+1.3)	-108.4 (+139.3)	179.1 (-0.7)	31.0 (-0.2)	111.9 (-0.1)	14.7 (-166.7)	148.6 (+0.6)

Table S7: Thermochemical and geometrical features of the different conformers of the closed form of compound **3a**, as evaluated at the ωB97X-D/6-311G(d)/IEF-PCM(acetonitrile) level of theory: relative Gibbs free energy (ΔG^0, kJ mol^{-1}), corresponding MB population (P, %) at 298.15 K and torsional angles (θ, °, with the relative difference with respect to the most stable conformer in parentheses).

	ΔG^0	P	θ_A	θ_B	θ_{Pt}	$\theta_{A'}$	$\theta_{B'}$	$\theta_{Pt'}$	$\theta_{A''}$	$\theta_{B''}$	$\theta_{Pt''}$
						**CCO**					
1	0.0	37.6	113.2	-176.5	-29.6	3.3	-0.6	28.8	111.9	-178.2	148.2
2	0.6	29.4	112.6 (-0.6)	-179.3 (-2.9)	31.2 (+60.8)	2.2 (-1.1)	-1.2 (-0.6)	28.4 (-0.4)	113.1 (+1.2)	-179.0 (-0.8)	-147.8 (+63.9)
3	3.7	8.5	112.7 (-0.5)	-177.3 (-0.8)	-30.0 (-0.4)	2.0 (-1.3)	0.5 (+1.0)	-28.3 (-57.1)	111.8 (-0.1)	-178.3 (-0.1)	148.4 (+0.1)
4	4.3	6.7	112.1 (-1.1)	179.6 (-3.9)	30.9 (+60.5)	1.9 (-1.4)	0.9 (+1.5)	-29.1 (-57.9)	112.5 (+0.6)	-179.4 (-1.2)	-148.4 (+63.4)
5	4.4	6.4	113.0 (-0.2)	-178.5 (-2.1)	32.0 (+61.6)	3.8 (+0.5)	-0.3 (+0.3)	30.3 (+1.5)	111.7 (-0.2)	-178.6 (-0.4)	147.8 (-0.4)
6	5.5	4.1	112.5 (-0.7)	179.8 (-3.8)	30.7 (+60.3)	2.8 (-0.5)	1.5 (+2.1)	-27.7 (-56.5)	112.0 (+0.1)	-178.1 (+0.1)	147.9 (-0.4)
7	6.6	2.7	111.9 (-1.3)	-178.9 (-2.4)	-32.0 (-2.4)	-163.5 (-166.8)	3.2 (+3.8)	-30.3 (-59.1)	112.2 (+0.2)	-177.9 (+0.3)	148.3 (+0.1)
8	7.6	1.8	112.5 (-0.7)	-177.1 (-0.6)	-30.8 (-1.3)	-161.5 (-164.9)	2.0 (+2.5)	30.0 (+1.2)	112.6 (+0.6)	-179.8 (-1.6)	-148.8 (+62.9)
9	7.6	1.8	112.5 (-0.7)	-177.1 (-0.6)	-30.8 (-1.3)	-161.5 (-164.9)	2.0 (+2.5)	30.0 (+1.2)	112.6 (+0.6)	-179.8 (-1.6)	-148.8 (+62.9)
10	10.7	0.5	112.2 (-1.0)	179.8 (-3.8)	31.0 (+60.6)	-161.5 (-164.8)	5.6 (+6.2)	-27.5 (-56.4)	112.5 (+0.5)	-179.7 (-1.5)	-148.4 (+63.4)
11	11.5	0.4	112.6 (-0.6)	-177.1 (-0.7)	-30.2 (-0.6)	-160.1 (-163.5)	3.4 (+4.0)	29.7 (+0.9)	112.3 (+0.4)	-177.9 (+0.3)	147.9 (-0.3)
12	11.8	0.3	112.6 (-0.6)	-177.3 (-0.8)	-30.6 (-1.0)	-162.0 (-165.3)	5.3 (+5.9)	-28.9 (-57.7)	112.2 (+0.3)	179.9 (-1.9)	-148.6 (+63.1)
						**COO**					
1	0.0	28.8	3.2	-0.5	29.4	2.1	-1.2	28.6	112.8	-179.5	31.4
2	0.9	19.9	2.3 (-0.9)	2.5 (+3.0)	-29.4 (-58.8)	2.4 (+0.4)	1.7 (+2.9)	-29.9 (-58.5)	111.6 (-1.2)	178.3 (-2.2)	30.0 (-1.3)
3	1.0	19.2	2.3 (-0.9)	-2.1 (-1.5)	28.4 (-1.0)	2.5 (+0.4)	-2.1 (-0.9)	29.0 (+0.4)	112.5 (-0.4)	-177.9 (+1.6)	-31.2 (-62.6)
4	1.6	15.0	3.6 (+0.4)	1.4 (+1.9)	-29.3 (-58.8)	3.4 (+1.3)	1.9 (+3.1)	-27.2 (-55.8)	112.0 (-0.8)	-178.8 (+0.7)	-31.5 (-62.9)
5	2.1	12.6	2.8 (-0.4)	-1.8 (-1.3)	29.2 (-0.2)	3.1 (+1.0)	3.5 (+4.7)	-28.6 (-57.2)	113.0 (+0.2)	-178.9 (+0.6)	32.1 (+0.7)
6	4.6	4.5	2.4 (-0.8)	-1.4 (-0.9)	28.9 (-0.5)	3.0 (+1.0)	1.8 (+3.0)	-28.1 (-56.7)	112.4 (-0.4)	-177.1 (+2.4)	-31.0 (-62.4)
						**OOO**					
1	0.0	51.2	-2.6	1.9	-30.8	-2.6	0.2	-29.5	-2.8	-2.9	28
2	1.4	29.1	-3.5 (-0.9)	-2.7 (-4.6)	28.9 (+59.7)	-2.4 (+0.2)	-0.5 (-0.7)	30.3 (+59.7)	-2.4 (+0.4)	-0.8 (+2.1)	30.2 (+2.2)
3	4.5	8.3	-3.7 (-1.1)	0.8 (-1.1)	-30.9 (-0.1)	-3.0 (-0.4)	-2.1 (-2.4)	28.7 (+58.2)	-2.6 (+0.2)	-2.0 (+0.9)	28.8 (+0.9)
4	5.9	4.8	-3.8 (-1.2)	0.5 (-1.4)	-31.3 (-0.5)	-2.8 (-0.2)	1.9 (+1.7)	-30.2 (-0.7)	-3.2 (-0.4)	0.7 (+3.6)	-30.8 (-58.8)
5	6.4	3.8	-3.6 (-1.0)	-2.6 (-4.5)	29.1 (+59.9)	161.7 (+164.4)	-5.3 (-5.5)	29.9 (+59.4)	-2.4 (+0.4)	2.6 (+5.5)	-28.5 (-56.4)
6	8.2	1.9	-2.0 (+0.6)	0.0 (-1.8)	30.6 (+61.4)	-3.1 (-0.4)	-2.2 (-2.4)	28.7 (+58.2)	162.3 (+165.1)	-1.5 (+1.4)	-29.1 (-57.1)
7	9.9	1.0	-1.6 (+1.1)	0.8 (-1.1)	31.2 (+62.0)	-2.6 (+0.0)	-0.7 (-0.9)	30.3 (+59.8)	163.6 (+166.4)	-4.3 (-1.4)	30.2 (+2.2)

Table S8: Thermochemical and geometrical features of the different conformers of the open forms of compound **3a**, as evaluated at the ωB97X-D/6-311G(d)/IEF-PCM(acetonitrile) level of theory: relative Gibbs free energy (ΔG^0, kJ mol^{-1}), corresponding MB population (P, %) at 298.15 K and torsional angles (θ, °, with the relative difference with respect to the most stable conformer in parentheses).

3a

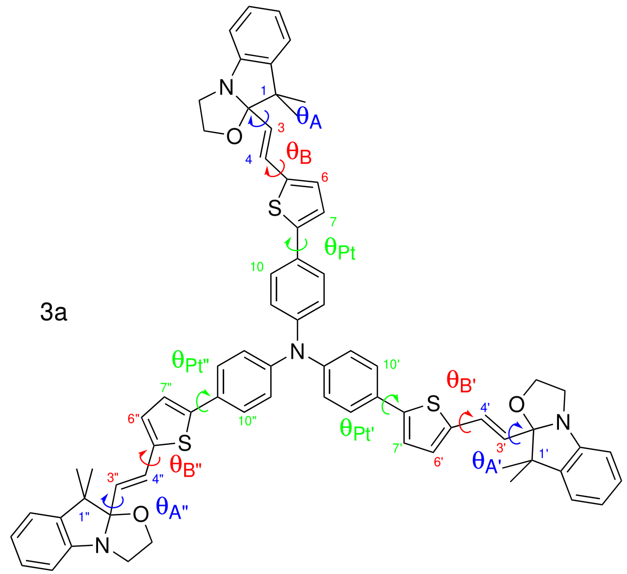

	CCC			CCO			COO			OOO		
	$BLA_{2,5}$	$BLA_{2',5'}$	$BLA_{2'',5''}$	$BLA_{2,5}$	$BLA_{2',5'}$	$BLA_{2'',5''}$	$BLA_{2,5}$	$BLA_{2',5'}$	$BLA_{2'',5''}$	$BLA_{2,5}$	$BLA_{2',5'}$	$BLA_{2'',5''}$
1	0.147	0.147	0.147	0.147	0.059	0.147	0.061	0.061	0.147	0.062	0.062	0.062
2	0.147	0.147	0.147	0.147	0.059	0.147	0.061	0.061	0.147	0.062	0.062	0.062
3	0.147	0.147	0.147	0.147	0.059	0.147	0.061	0.061	0.147	0.063	0.062	0.062
4	0.147	0.147	0.147	0.147	0.060	0.147	0.061	0.061	0.147	0.063	0.062	0.063
5	0.146	0.147	0.149	0.147	0.059	0.147	0.061	0.061	0.147	0.062	0.063	0.062
6	0.147	0.147	0.147	0.147	0.059	0.147	0.061	0.061	0.147	0.062	0.062	0.063
7	0.147	0.147	0.147	0.147	0.060	0.147				0.062	0.062	0.062
8	0.147	0.147	0.147	0.147	0.061	0.147						
9	0.149	0.147	0.147	0.147	0.061	0.147						
10	0.149	0.147	0.147	0.147	0.060	0.147						
11	0.147	0.147	0.149	0.147	0.061	0.147						
12	0.147	0.147	0.147	0.147	0.060	0.147						
13	0.150	0.149	0.150									
14	0.149	0.147	0.147									
15	0.149	0.147	0.147									
16	0.149	0.149	0.147									
17	0.149	0.147	0.149									
18	0.149	0.147	0.147									
19	0.149	0.149	0.149									
20	0.149	0.147	0.147									

Table S9: Bond length alternation (BLA, Å) of the different conformers of compound **3a**, as evaluated at the ωB97X-D/6-311G(d)/IEF-PCM(acetonitrile) level of theory.

3b

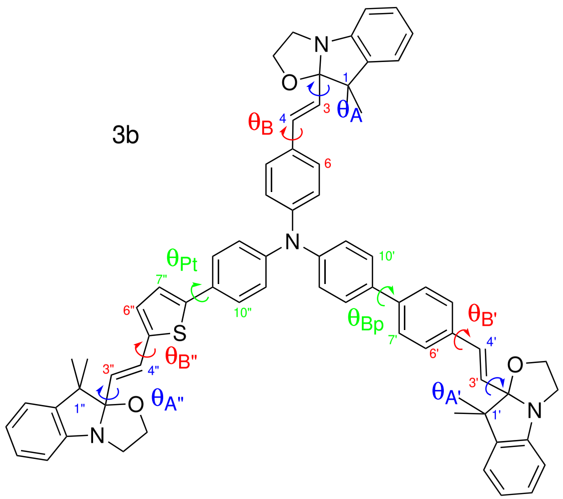

	ΔG^0	P	θ_A	θ_B	$\theta_{A'}$	$\theta_{B'}$	$\theta_{Bp'}$	$\theta_{A"}$	$\theta_{B"}$	θ_{PhTh}	$BLA_{2,5}$	$BLA_{2',5'}$	$BLA_{2",5"}$
							**CCC** [Ph(C)-BiPh(C)-PhTh(C)]						
1	0.0	28.6	-111.2	172.1	-112.6	166.7	-140.7	-112.5	179.7	148.300	0.156	0.157	0.147
2	1.2	17.3	109.3 (-139.5)	174.5 (+2.4)	109.5 (-137.9)	-168.4 (+24.9)	-140.4 (+0.3)	-113.0 (-0.5)	178.6 (-1.1)	147.8 (-0.5)	0.156	0.157	0.147
3	1.5	15.8	-111.8 (-0.6)	167.3 (-4.8)	-112.0 (+0.7)	171.0 (+4.3)	-140.4 (+0.4)	108.3 (-139.2)	-179.9 (+0.4)	147.6 (-0.7)	0.156	0.157	0.147
4	1.6	14.9	-111.9 (-0.7)	168.8 (-3.3)	109.3 (-138.0)	-175.7 (+17.6)	-140.8 (-0.1)	109.2 (-138.2)	-177.6 (+2.7)	149.2 (+0.9)	0.156	0.157	0.147
5	2.2	11.9	-111.6 (-0.4)	169.4 (-2.7)	109.2 (-138.1)	-172.8 (+20.5)	-140.7 (+0.0)	-112.2 (+0.3)	-179.7 (+0.6)	148.8 (+0.5)	0.156	0.157	0.147
6	3.1	8.3	109.5 (-139.3)	175.9 (+3.8)	-111.8 (+0.8)	169.3 (+2.5)	-140.3 (+0.5)	108.5 (-138.9)	-179.2 (+1.1)	148.5 (+0.2)	0.156	0.157	0.147
7	5.4	3.3	-112.2 (-0.9)	166.9 (-5.1)	-112.5 (+0.2)	167.8 (+1.1)	-140.5 (+0.2)	-111.6 (+0.9)	10.9 (-168.7)	148.9 (+0.6)	0.156	0.157	0.149

Table S10: Thermochemical and geometrical features of the different conformers of the closed form of compound **3b**, as evaluated at the ωB97X-D/6-311G(d)/IEF-PCM(acetonitrile) level of theory: relative Gibbs free energy (ΔG^0, kJ mol^{-1}), corresponding MB population (P, %) at 298.15 K, torsional angles (θ, °, with the relative difference with respect to the most stable conformer in parentheses) and bond length alternation (BLA, Å).

	ΔG^0	P	θ_A	θ_B	$\theta_{A'}$	$\theta_{B'}$	θ_{Bp}	$\theta_{A"}$	$\theta_{B"}$	θ_{Pt}	$BLA_{2,5}$	$BLA_{2',5'}$	$BLA_{2",5"}$
						**CCO** [Ph(C)-BiPh(C)-PhTh(O)]							
1	0.0	46.3	-111.7	168.6	-112.2	170.1	-140.1	28	-178.2	153	0.157	0.157	0.056
2	1.0	31.5	109.6 (-138.7)	180.0 (+11.3)	-112.1 (+0.1)	168.1 (-2.0)	-140.0 (+0.0)	28.4 (+0.4)	-177.8 (+0.4)	154.6 (+1.5)	0.157	0.157	0.056
3	3.3	12.4	-112.0 (-0.3)	167.3 (-1.3)	109.1 (-138.7)	-176.8 (+13.1)	-140.5 (-0.4)	28.0 (+0.0)	-178.0 (+0.2)	153.5 (+0.5)	0.156	0.157	0.056
4	4.0	9.1	109.7 (-138.6)	176.9 (+8.2)	109.4 (-138.4)	-170.0 (+19.9)	-140.4 (-0.3)	26.1 (-1.8)	-179.4 (-1.2)	151.7 (-1.4)	0.157	0.157	0.056
5	10.3	0.7	-112.9 (-1.3)	164.7 (-4.0)	-111.7 (+0.4)	169.4 (-0.7)	-139.9 (+0.2)	27.6 (-0.3)	1.9 (-179.9)	151.2 (-1.8)	0.156	0.157	0.059
						**COC** [Ph(C)-BiPh(O)-PhTh(C)]							
1	0.0	46.8	109.3	-175.4	1.5	177.3	-143.2	109.1	-177.9	148.9	0.156	0.084	0.147
2	2.4	18.0	109.6 (+0.3)	173.9 (-10.6)	1.9 (+0.5)	176.4 (-0.9)	-142.7 (+0.5)	-112.3 (+138.6)	-179.6 (-1.7)	148.7 (-0.2)	0.156	0.085	0.147
3	2.5	17.0	-112.4 (+138.3)	166.8 (-17.7)	4.0 (+2.5)	-177.1 (+5.6)	-140.7 (+2.5)	109.3 (+0.1)	-176.6 (+1.3)	149.6 (+0.8)	0.156	0.085	0.147
4	2.6	16.6	-112.7 (+138.0)	167.4 (-17.2)	2.7 (+1.3)	177.3 (-0.0)	-142.6 (+0.6)	-112.7 (+138.2)	179.3 (-2.7)	147.9 (-0.9)	0.156	0.085	0.147
5	8.2	1.7	-112.3 (+138.4)	166.9 (-17.7)	3.5 (+2.0)	-177.4 (+5.3)	-141.3 (+2.0)	-112.0 (+138.8)	-9.1 (+168.8)	148.8 (-0.1)	0.156	0.085	0.149
						**OCC** [Ph(O)-BiPh(C)-PhTh(C)]							
1	0.0	62.1	1.8	177.9	-112	166.9	-140.1	-111.9	-179	149.2	0.064	0.157	0.147
2	3.9	12.7	4.0 (+2.2)	-178.8 (+3.3)	109.2 (-138.8)	176.8 (+9.9)	-139.9 (+0.1)	-112.0 (-0.2)	-179.7 (-0.7)	149.2 (+0.0)	0.065	0.157	0.147
3	4.4	10.5	3.4 (+1.6)	177.9 (+0.0)	109.3 (-138.7)	175.0 (+8.2)	-140.3 (-0.2)	106.1 (-142.0)	-178.5 (+0.5)	147.8 (-1.4)	0.065	0.157	0.147
4	4.6	9.7	4.4 (+2.6)	-179.4 (+2.7)	-112.7 (-0.7)	167.8 (+1.0)	-140.2 (-0.1)	-112.2 (-0.3)	-12.1 (+166.9)	149.0 (-0.2)	0.065	0.157	0.150
5	6.2	5.0	1.1 (-0.7)	176.2 (-1.7)	-112.0 (+0.0)	167.6 (+0.7)	-140.3 (-0.2)	108.6 (-139.5)	-178.7 (+0.3)	148.0 (-1.2)	0.064	0.157	0.147

Table S11: Thermochemical and geometrical features of the different conformers of the first open form of compound **3b**, as evaluated at the ωB97X-D/6-311G(d)/IEF-PCM(acetonitrile) level of theory: relative Gibbs free energy (ΔG^0, kJ mol^{-1}), corresponding MB population (P, %) at 298.15 K, torsional angles (θ, °, with the relative difference with respect to the most stable conformer in parentheses) and bond length alternation (BLA, Å).

	ΔG^0	P	θ_A	θ_B	$\theta_{A'}$	$\theta_{B'}$	θ_{Bp}	$\theta_{A"}$	$\theta_{B"}$	θ_{Pt}	$BLA_{2,5}$	$BLA_{2',5'}$	$BLA_{2",5"}$
**COO** [Ph(C)-BiPh(O)-PhTh(O)]													
1	0.0	49.2	-111.5	169	3.2	-179.5	-140.6	1.9	179.8	150.8	0.157	0.086	0.057
2	1.4	27.8	-112.8 (-1.3)	164.8 (-4.2)	2.9 (-0.3)	-179.3 (+0.3)	-140.9 (-0.4)	2.9 (+1.0)	0.8 (-179.0)	151.6 (+0.7)	0.157	0.086	0.060
3	1.9	23.0	109.5 (-138.9)	-179.6 (+11.4)	3.5 (+0.3)	-177.8 (+1.7)	-141.1 (-0.5)	2.5 (+0.6)	-179.9 (+0.3)	151.3 (+0.4)	0.157	0.086	0.057
**OCO** [Ph(O)-BiPh(C)-PhTh(O)]													
1	0.0	90.5	2.9	178.3	-112	167.8	-140.2	2.4	-179.5	148.9	0.069	0.157	0.061
2	6.7	6.0	3.8 (+0.9)	179.3 (+1.0)	109.4 (-138.7)	-175.9 (+16.3)	-140.1 (+0.0)	2.3 (-0.1)	-178.9 (+0.6)	149.8 (+0.8)	0.069	0.157	0.061
3	8.1	3.5	3.9 (+1.0)	-179.7 (+2.1)	-112.4 (-0.4)	167.3 (-0.5)	-140.0 (+0.2)	2.6 (+0.2)	-0.1 (+179.4)	148.6 (-0.4)	0.069	0.157	0.064
**OOC** [Ph(O)-BiPh(O)-PhTh(C)]													
1	0.0	81.9	3.4	179.3	2.2	178.8	-140.5	-112.9	179.4	148.5	0.067	0.087	0.147
2	5.4	9.3	3.4 (+0.1)	179.2 (-0.1)	2.3 (+0.1)	178.7 (-0.1)	-140.7 (-0.1)	-111.5 (+1.4)	-3.2 (+177.4)	149.7 (+1.2)	0.066	0.087	0.149
3	5.5	8.7	3.2 (-0.1)	179.5 (+0.2)	2.4 (+0.1)	177.3 (-1.5)	-141.3 (-0.8)	108.3 (-138.8)	-179.4 (+1.2)	147.8 (-0.7)	0.067	0.087	0.147
**OOO** [Ph(O)-BiPh(O)-PhTh(O)]													
1	0.0	93.8	2.9	178.3	2.1	178	-140.9	2.1	166.7	150.5	0.070	0.087	0.062
2	6.7	6.2	2.8 (-0.1)	178.3 (+0.0)	2.8 (+0.7)	-178.5 (+3.6)	-140.5 (+0.4)	3.3 (+1.2)	-6.6 (-173.3)	149.0 (-1.5)	0.070	0.087	0.064

Table S12: Thermochemical and geometrical features of the different conformers of the two and three times open form of compound **3b**, as evaluated at the ωB97X-D/6-311G(d)/IEF-PCM(acetonitrile) level of theory: relative Gibbs free energy (ΔG^0, kJ mol^{-1}), corresponding MB population (P, %) at 298.15 K, torsional angles (θ, °, with the relative difference with respect to the most stable conformer in parentheses) and bond length alternation (BLA, Å).

S2 NLO properties

	C				O			
	Static	1907 nm	1300 nm	1064 nm	Static	1907 nm	1300 nm	1064 nm
1	0.4 (4.11)	0.3 (3.86)	0.3 (3.67)	0.3 (3.46)	17.1 (4.80)	14.2 (4.88)	24.0 (4.93)	50.0 (4.97)
2	0.1 (1.70)	0.1 (2.00)	0.1 (2.61)	0.2 (3.13)	16.0 (4.83)	13.1 (4.90)	21.9 (4.95)	45.3 (4.97)
3	0.6 (4.97)	0.4 (4.59)	0.5 (4.56)	0.5 (4.49)	34.7 (4.88)	29.8 (4.94)	60.1 (4.97)	211.4 (4.97)
4	0.4 (3.52)	0.3 (3.49)	0.3 (3.54)	0.4 (3.57)	18.1 (4.82)	15.1 (4.89)	26.0 (4.95)	55.7 (5.00)
5	2.6 (5.21)	2.1 (5.24)	2.7 (5.22)	3.6 (5.20)	16.7 (4.88)	13.9 (4.93)	23.2 (4.97)	47.2 (5.00)
6	2.2 (5.82)	1.8 (5.74)	2.3 (5.65)	3.0 (5.55)	17.7 (4.84)	14.6 (4.90)	25.1 (4.96)	53.7 (5.00)
7	1.9 (5.27)	1.6 (5.23)	2.1 (5.23)	2.7 (5.22)	36.3 (4.88)	31.6 (4.94)	65.1 (4.98)	238.6 (5.02)
8	2.3 (4.91)	1.9 (4.97)	2.5 (5.00)	3.2 (5.03)	15.3 (4.94)	12.6 (4.97)	21.0 (4.99)	42.1 (5.01)
9					33.5 (4.92)	28.8 (4.96)	57.2 (4.99)	189.2 (5.00)
Avg.	0.5 (3.78)	0.4 (3.67)	0.5 (3.71)	0.6 (3.71)	20.0 (4.82)	16.7 (4.89)	29.9 (4.94)	77.3 (4.97)

Table S13: Computed static and dynamic first hyperpolarizabilities (β_{HRS} in 10^3 a.u., DR in parentheses) for the different conformers of compound **1**, as evaluated at the TDDFT/M06-2X/6-311+G(d)/IEF-PCM (acetonitrile) level of approximation. The last line contains averaged values using the MB population at 298.15 K as calculated at the ωB97X-D/6-311G(d)/IEF-PCM (acetonitrile) level of theory.

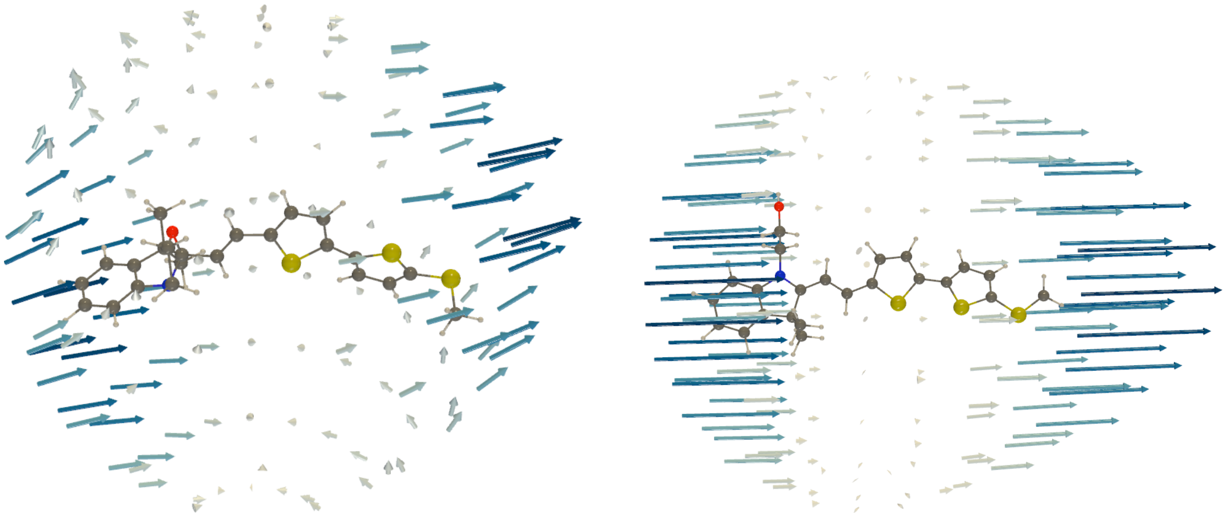

(a) **C**: $\beta_{HRS} = 0.3$ (3.86), sc=5×10^{-3}

(b) **O**: $\beta_{HRS} = 14.2$ (4.88), sc=10^{-4}

Figure S1: USR (together with β_{HRS} [10^3 a.u., DR in parentheses] and scaling factor [sc, Å a.u.$^{-1}$]) of the dynamic ($\lambda = 1907$ nm) β tensor of the most stable conformers of each form of **1**, as evaluated at the TDDFT/M06-2X/6-311+G(d)/IEF-PCM (acetonitrile) level of approximation.

	**CC**				**CO**				**OO**			
	Static	1907 nm	1300 nm	1064 nm	Static	1907 nm	1300 nm	1064 nm	Static	1907 nm	1300 nm	1064 nm
1	0.5 (4.08)	0.4 (3.78)	0.5 (3.83)	0.6 (3.89)	26.1 (4.80)	25.3 (4.89)	48.9 (4.94)	140.8 (4.97)	3.1 (2.35)	2.6 (2.46)	4.5 (2.59)	13.3 (2.32)
2	0.5 (6.02)	0.5 (5.46)	0.6 (5.38)	0.7 (5.25)	26.5 (4.80)	25.6 (4.89)	50.0 (4.95)	147.3 (4.98)	2.0 (2.54)	1.7 (2.66)	3.1 (2.72)	10.8 (2.16)
3	0.2 (3.68)	0.2 (3.83)	0.2 (3.93)	0.3 (4.01)	28.9 (4.86)	27.9 (4.93)	54.2 (4.96)	161.0 (4.98)	2.6 (1.95)	2.2 (2.03)	4.5 (2.29)	24.1 (2.44)
4	0.2 (3.45)	0.2 (3.27)	0.2 (3.31)	0.2 (3.33)	27.7 (4.80)	26.9 (4.88)	53.2 (4.95)	159.5 (5.01)				
5	0.3 (3.20)	0.2 (3.29)	0.3 (3.34)	0.3 (3.37)	28.7 (4.84)	27.5 (4.91)	53.5 (4.95)	159.4 (4.97)				
6	0.5 (3.64)	0.4 (3.70)	0.4 (3.77)	0.5 (3.86)	31.4 (4.86)	30.6 (4.92)	61.3 (4.97)	194.5 (5.01)				
7	0.6 (6.27)	0.6 (5.65)	0.7 (5.51)	1.0 (5.36)	27.5 (4.80)	26.7 (4.89)	53.0 (4.96)	159.2 (5.01)				
8	0.8 (4.92)	0.7 (4.64)	0.8 (4.67)	1.0 (4.70)	30.0 (4.84)	29.0 (4.91)	57.3 (4.96)	173.5 (5.01)				
9					24.8 (4.86)	24.2 (4.92)	45.6 (4.97)	118.7 (5.00)				
10					25.5 (4.87)	24.9 (4.93)	47.4 (4.97)	128.3 (5.00)				
11					27.7 (4.93)	27.0 (4.97)	51.2 (4.99)	138.9 (5.00)				
Avg.	0.4 (4.01)	0.3 (3.86)	0.4 (3.91)	0.5 (3.96)	27.0 (4.81)	26.1 (4.90)	50.8 (4.95)	148.5 (4.98)	2.8 (2.33)	2.3 (2.43)	4.2 (2.57)	14.4 (2.31)

Table S14: Computed static and dynamic first hyperpolarizabilities (β_{HRS} in 10^3 a.u., DR in parentheses) for the different conformers of compound **2a**, as evaluated at the TDDFT/M06-2X/6-311+G(d)/IEF-PCM (acetonitrile) level of approximation. The last line contains averaged values using the MB population at 298.15 K as calculated at the ωB97X-D/6-311G(d)/IEF-PCM (acetonitrile) level of theory.

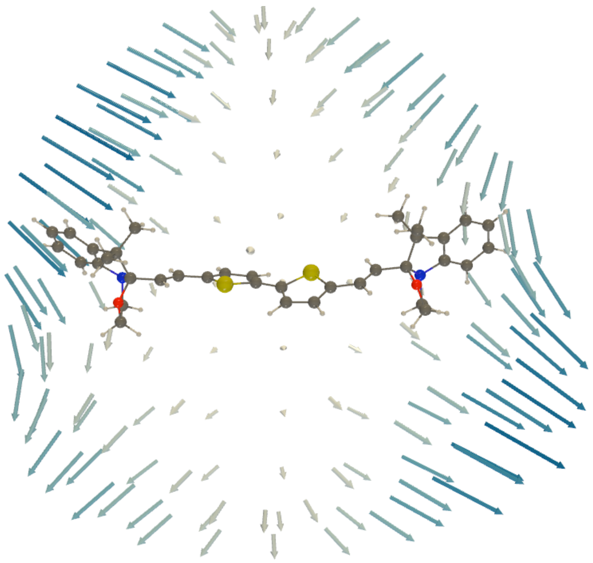

(a) **CC**: $\beta_{HRS} = 0.4$ (3.78), sc=5×10^{-3}

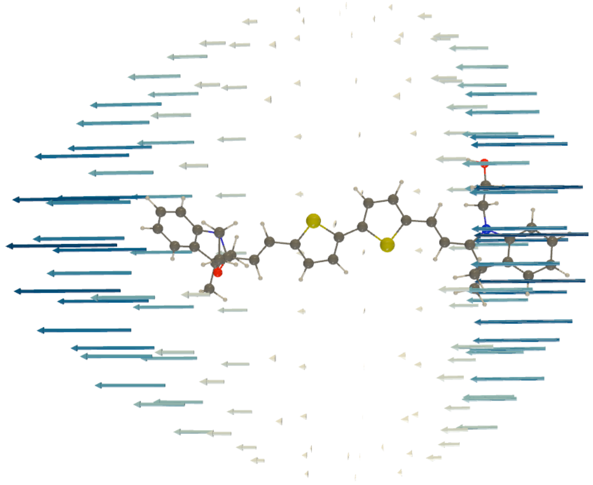

(b) **CO**: $\beta_{HRS} = 25.3$ (4.89), sc=10^{-4}

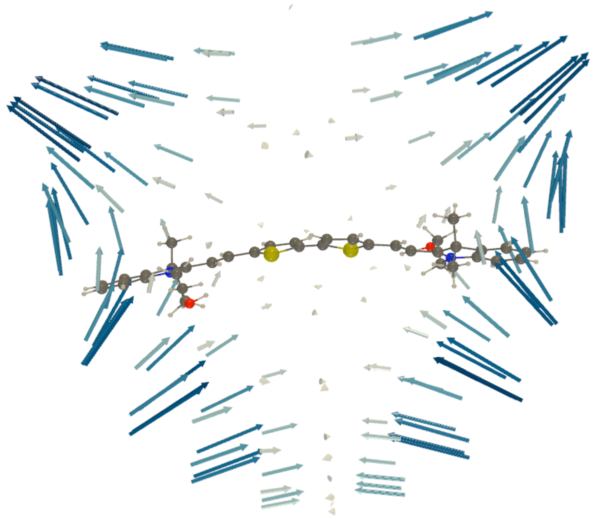

(c) **OO**: $\beta_{HRS} = 2.6$ (2.46), sc=10^{-3}

Figure S2: USR (together with β_{HRS} [10^3 a.u., DR in parentheses] and scaling factor [sc, Å a.u.$^{-1}$]) of the dynamic ($\lambda = 1907$ nm) β tensor of the most stable conformers of each form of **2a**, as evaluated at the TDDFT/M06-2X/6-311+G(d)/IEF-PCM (acetonitrile) level of approximation.

	CC				CO				OO			
	Static	1907 nm	1300 nm	1064 nm	Static	1907 nm	1300 nm	1064 nm	Static	1907 nm	1300 nm	1064 nm
1	0.5 (5.13)	0.3 (3.95)	0.4 (4.00)	0.4 (4.03)	6.9 (4.49)	6.1 (4.62)	8.9 (4.75)	14.4 (4.87)	4.9 (2.38)	4.3 (2.43)	6.2 (2.50)	9.7 (2.59)
2	0.8 (5.59)	0.6 (4.48)	0.7 (4.55)	0.7 (4.61)	6.8 (4.41)	6.0 (4.55)	8.8 (4.69)	14.1 (4.83)	4.7 (2.53)	4.1 (2.55)	5.8 (2.60)	9.1 (2.66)
3	0.8 (5.27)	0.6 (4.28)	0.7 (4.32)	0.8 (4.36)	6.8 (4.42)	6.0 (4.60)	8.7 (4.72)	13.7 (4.85)	4.2 (2.71)	3.7 (2.70)	5.1 (2.76)	8.0 (2.77)
4	0.6 (4.37)	0.4 (3.60)	0.5 (3.67)	0.5 (3.72)	7.2 (4.49)	6.4 (4.60)	9.3 (4.73)	14.9 (4.85)	4.8 (2.45)	4.3 (2.52)	6.2 (2.58)	9.7 (2.66)
5	0.5 (3.61)	0.4 (3.29)	0.4 (3.34)	0.5 (3.37)	6.7 (4.52)	6.0 (4.65)	8.8 (4.76)	14.1 (4.88)				
6	0.3 (4.96)	0.3 (3.85)	0.3 (3.84)	0.3 (3.80)								
7	0.4 (5.80)	0.3 (4.09)	0.3 (4.09)	0.4 (4.06)								
Avg.	0.5 (5.11)	0.4 (3.99)	0.4 (4.04)	0.5 (4.07)	6.9 (4.48)	6.1 (4.61)	8.9 (4.74)	14.4 (4.87)	4.9 (2.43)	4.2 (2.47)	6.1 (2.54)	9.5 (2.62)

Table S15: Computed static and dynamic first hyperpolarizabilities (β_{HRS} in 10^3 a.u., DR in parentheses) for the different conformers of compound **2b**, as evaluated at the TDDFT/M06-2X/6-311+G(d)/IEF-PCM (acetonitrile) level of approximation. The last line contains averaged values using the MB population at 298.15 K as calculated at the ωB97X-D/6-311G(d)/IEF-PCM (acetonitrile) level of theory.

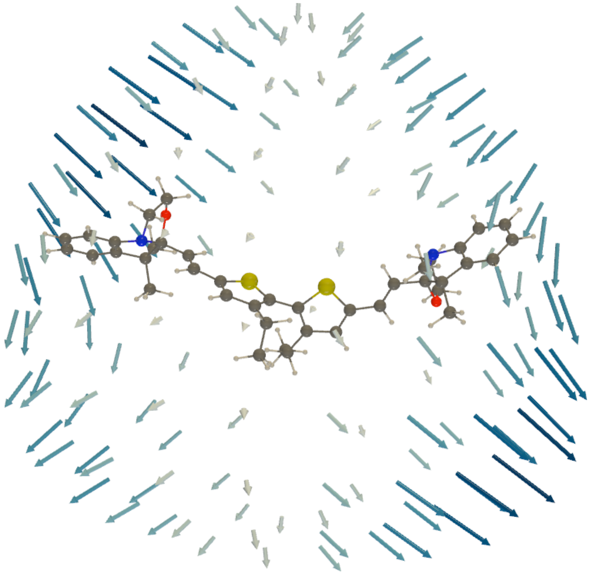

(a) **CC**: $\beta_{HRS} = 0.3$ (3.95), sc=5×10^{-3}

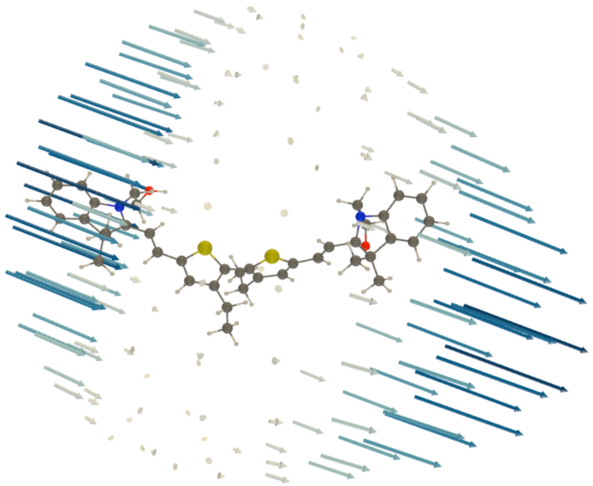

(b) **CO**: $\beta_{HRS} = 6.1$ (4.62), sc=5×10^{-4}

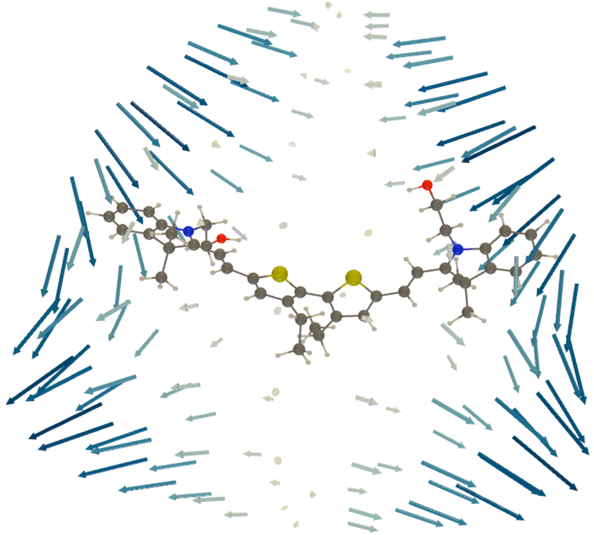

(c) **OO**: $\beta_{HRS} = 4.3$ (2.43), sc=5×10^{-3}

Figure S3: USR (together with β_{HRS} [10^3 a.u., DR in parentheses] and scaling factor [sc, Å a.u.$^{-1}$]) of the dynamic ($\lambda = 1907$ nm) β tensor of the most stable conformers of each form of **2b**, as evaluated at the TDDFT/M06-2X/6-311+G(d)/IEF-PCM (acetonitrile) level of approximation.

	CC				CO				OO			
	Static	1907 nm	1300 nm	1064 nm	Static	1907 nm	1300 nm	1064 nm	Static	1907 nm	1300 nm	1064 nm
1	1.5 (3.32)	1.2 (3.06)	1.5 (3.09)	2.0 (3.11)	37.0 (4.78)	32.1 (4.88)	72.8 (4.96)	623.9 (5.02)	12.2 (2.35)	10.7 (2.49)	24.8 (2.54)	105.0 (1.07)
2	1.5 (3.31)	1.2 (3.06)	1.5 (3.10)	2.0 (3.11)	37.4 (4.79)	32.4 (4.88)	73.4 (4.96)	646.2 (5.02)	7.6 (2.69)	7.1 (2.81)	16.5 (2.51)	60.0 (1.16)
3	1.4 (3.05)	1.1 (2.90)	1.4 (2.94)	1.9 (2.95)	41.0 (4.81)	35.8 (4.89)	81.6 (4.97)	771.7 (5.02)	10.3 (2.50)	9.2 (2.64)	21.5 (2.55)	82.0 (1.07)
4	1.3 (3.52)	1.0 (3.47)	1.3 (3.54)	1.8 (3.57)	39.5 (4.81)	34.5 (4.90)	80.4 (4.97)	873.7 (5.01)				
5	1.4 (3.50)	1.1 (3.28)	1.4 (3.32)	1.9 (3.34)	39.3 (4.80)	33.9 (4.88)	76.0 (4.96)	604.7 (5.02)				
6	1.3 (3.55)	1.1 (3.31)	1.4 (3.35)	1.8 (3.37)	42.7 (4.82)	37.3 (4.90)	87.2 (4.97)	1073.8 (5.01)				
7	0.9 (3.63)	0.7 (3.36)	0.9 (3.38)	1.2 (3.34)	40.8 (4.81)	35.3 (4.89)	81.3 (4.96)	811.0 (5.01)				
8	1.2 (3.79)	1.0 (3.37)	1.2 (3.37)	1.5 (3.34)	39.2 (4.81)	34.2 (4.89)	79.8 (4.96)	874.5 (5.01)				
9					37.2 (4.87)	32.5 (4.93)	73.3 (4.99)	608.7 (5.03)				
10					40.3 (4.86)	35.3 (4.93)	79.4 (4.99)	621.1 (5.03)				
Avg.	1.4 (3.30)	1.2 (3.08)	1.5 (3.12)	1.9 (3.13)	37.8 (4.79)	32.9 (4.88)	74.6 (4.96)	666.6 (5.02)	11.0 (2.44)	9.7 (2.58)	22.6 (2.54)	92.5 (1.09)

Table S16: Computed static and dynamic first hyperpolarizabilities (β_{HRS} in 10^3 a.u., DR in parentheses) for the different conformers of compound **2c**, as evaluated at the TDDFT/M06-2X/6-311+G(d)/IEF-PCM (acetonitrile) level of approximation. The last line contains averaged values using the MB population at 298.15 K as calculated at the ωB97X-D/6-311G(d)/IEF-PCM (acetonitrile) level of theory.

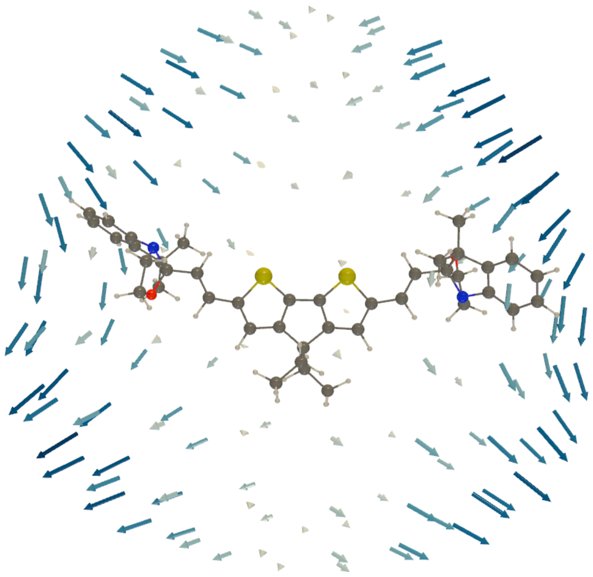
(a) **CC**: $\beta_{HRS} = 1.2$ (3.06), sc=10^{-3}

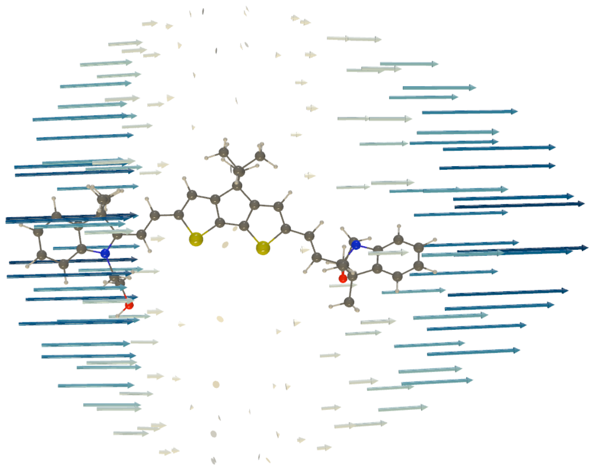
(b) **CO**: $\beta_{HRS} = 32.1$ (4.88), sc=10^{-4}

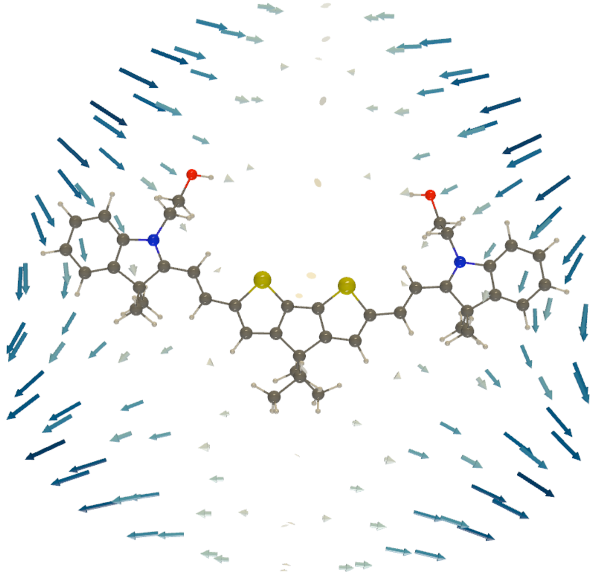
(c) **OO**: $\beta_{HRS} = 10.7$ (2.49), sc=10^{-4}

Figure S4: USR (together with β_{HRS} [10^3 a.u., DR in parentheses] and scaling factor [sc, Å a.u.$^{-1}$]) of the dynamic ($\lambda = 1907$ nm) β tensor of the most stable conformers of each form of **2c**, as evaluated at the TDDFT/M06-2X/6-311+G(d)/IEF-PCM (acetonitrile) level of approximation.

	CC				CO				OO			
	Static	1907 nm	1300 nm	1064 nm	Static	1907 nm	1300 nm	1064 nm	Static	1907 nm	1300 nm	1064 nm
1	0.4 (4.43)	0.4 (4.05)	0.4 (4.19)	0.5 (4.37)	34.2 (4.81)	31.6 (4.90)	75.1 (4.95)	1129.6 (4.93)	0.1 (3.99)	0.1 (3.35)	0.1 (2.83)	0.6 (3.09)
2	0.1 (2.16)	0.1 (2.62)	0.1 (2.85)	0.1 (3.00)	36.4 (4.81)	33.7 (4.90)	78.5 (4.95)	924.4 (4.93)	0.3 (2.36)	0.3 (2.60)	0.8 (2.73)	6.6 (2.04)
3					36.3 (4.93)	33.3 (4.97)	76.2 (5.00)	540.5 (5.01)	2.1 (2.87)	1.8 (2.73)	4.6 (2.92)	20.8 (1.14)
4					33.8 (4.81)	31.2 (4.90)	74.0 (4.95)	1075.6 (4.94)	1.8 (2.75)	1.3 (2.52)	3.0 (2.48)	15.3 (2.10)
5					35.6 (4.81)	32.6 (4.89)	75.9 (4.95)	885.1 (4.93)				
Avg.	0.4 (4.02)	0.3 (3.79)	0.4 (3.95)	0.5 (4.12)	34.9 (4.83)	32.1 (4.91)	75.7 (4.96)	1009.0 (4.94)	0.3 (3.75)	0.2 (3.23)	0.5 (2.82)	2.7 (2.85)

Table S17: Computed static and dynamic first hyperpolarizabilities (β_{HRS} in 10^3 a.u., DR in parentheses) for the different conformers of compound **2d**, as evaluated at the TDDFT/M06-2X/6-311+G(d)/IEF-PCM (acetonitrile) level of approximation. The last line contains averaged values using the MB population at 298.15 K as calculated at the ωB97X-D/6-311G(d)/IEF-PCM (acetonitrile) level of theory.

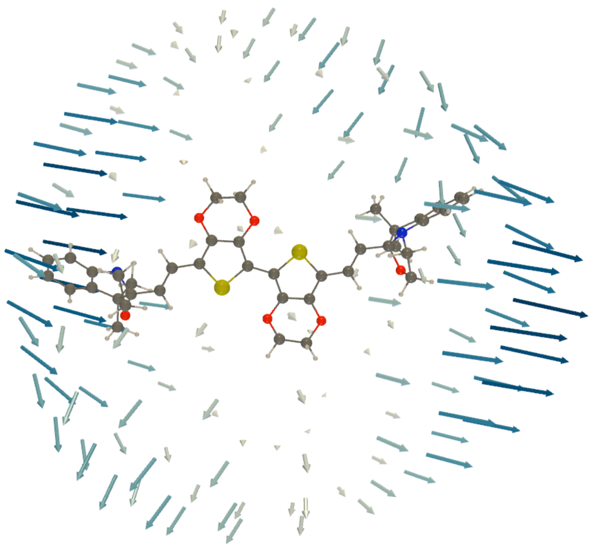

(a) **CC**: $\beta_{HRS} = 0.4$ (4.05), sc=5×10^{-3}

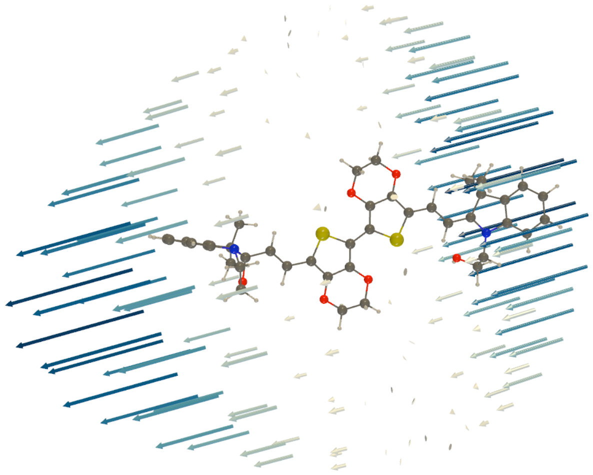

(b) **CO**: $\beta_{HRS} = 31.6$ (4.90), sc=10^{-4}

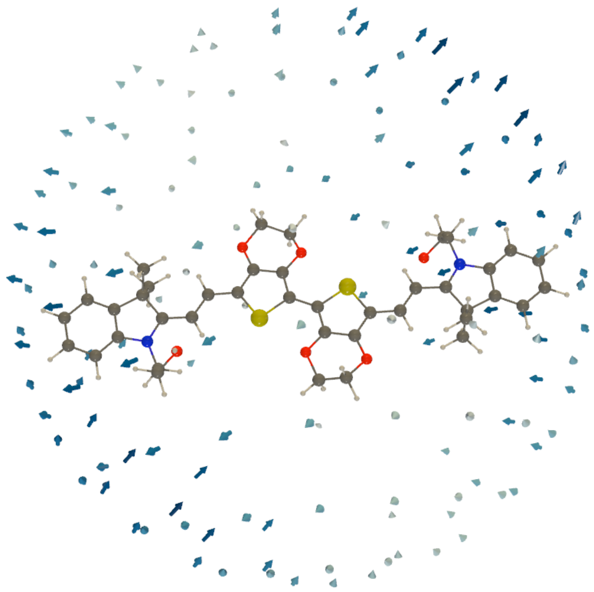

(c) **OO**: $\beta_{HRS} = 0.1$ (3.35), sc=10^{-2}

Figure S5: USR (together with β_{HRS} [10^3 a.u., DR in parentheses] and scaling factor [sc, Å a.u.$^{-1}$]) of the dynamic ($\lambda = 1907$ nm) β tensor of the most stable conformers of each form of **2d**, as evaluated at the TDDFT/M06-2X/6-311+G(d)/IEF-PCM (acetonitrile) level of approximation.

	CC				CO				OO			
	Static	1907 nm	1300 nm	1064 nm	Static	1907 nm	1300 nm	1064 nm	Static	1907 nm	1300 nm	1064 nm
1	1.5 (6.47)	1.3 (6.06)	2.0 (5.79)	3.4 (5.40)	57.3 (4.90)	62.0 (4.96)	178.2 (4.98)	1439.2 (4.95)	10.8 (2.70)	10.8 (2.64)	31.7 (2.74)	51.5 (0.13)
2	1.1 (3.16)	0.8 (3.46)	1.1 (3.45)	1.7 (3.41)	61.1 (4.90)	67.2 (4.96)	192.5 (4.98)	1567.8 (4.95)	16.2 (2.48)	14.6 (2.64)	41.0 (2.58)	66.6 (0.41)
3	1.4 (7.59)	1.0 (6.41)	1.2 (6.62)	1.7 (5.93)	53.9 (4.91)	58.2 (4.96)	163.7 (4.97)	1819.0 (4.92)	17.9 (2.67)	16.7 (2.85)	47.4 (2.89)	106.6 (1.14)
4	0.7 (5.02)	0.4 (6.15)	0.5 (6.07)	0.6 (3.63)	56.8 (4.90)	61.7 (4.96)	176.4 (4.98)	1552.4 (4.96)				
5	1.5 (7.00)	1.3 (6.02)	1.9 (5.83)	3.0 (5.42)	49.7 (4.96)	53.0 (5.00)	148.4 (4.97)	1471.2 (4.84)				
6	2.8 (5.37)	2.1 (5.20)	2.6 (5.20)	3.6 (4.92)	60.5 (4.90)	65.8 (4.95)	188.0 (4.98)	1543.5 (4.95)				
7	1.2 (3.38)	1.1 (4.23)	1.8 (4.48)	3.2 (4.68)	52.9 (5.02)	56.5 (5.03)	160.0 (4.98)	1191.2 (4.80)				
8	1.1 (2.39)	0.8 (2.66)	1.1 (2.74)	1.7 (2.79)	45.8 (5.10)	46.4 (5.10)	127.6 (5.03)	1201.5 (4.82)				
9	1.6 (3.82)	1.5 (4.16)	2.1 (4.21)	3.3 (4.25)	58.0 (4.91)	63.7 (4.96)	178.7 (4.98)	2015.2 (4.94)				
10	2.8 (4.72)	2.1 (4.30)	2.7 (4.32)	3.7 (4.15)	54.7 (4.90)	59.4 (4.95)	169.8 (4.97)	1554.9 (4.92)				
11	1.3 (3.62)	1.1 (4.09)	1.6 (4.09)	2.5 (4.08)	57.5 (4.90)	62.4 (4.95)	174.5 (4.97)	2014.6 (4.94)				
Avg.	1.4 (5.15)	1.1 (5.09)	1.6 (5.07)	2.5 (4.62)	56.4 (4.92)	61.1 (4.97)	174.2 (4.98)	1523.2 (4.93)	11.9 (2.66)	11.6 (2.65)	33.7 (2.72)	55.5 (0.20)

Table S18: Computed static and dynamic first hyperpolarizabilities (β_{HRS} in 10^3 a.u., DR in parentheses) for the different conformers of compound **2e**, as evaluated at the TDDFT/M06-2X/6-311+G(d)/IEF-PCM (acetonitrile) level of approximation. The last line contains averaged values using the MB population at 298.15 K as calculated at the ωB97X-D/6-311G(d)/IEF-PCM (acetonitrile) level of theory.

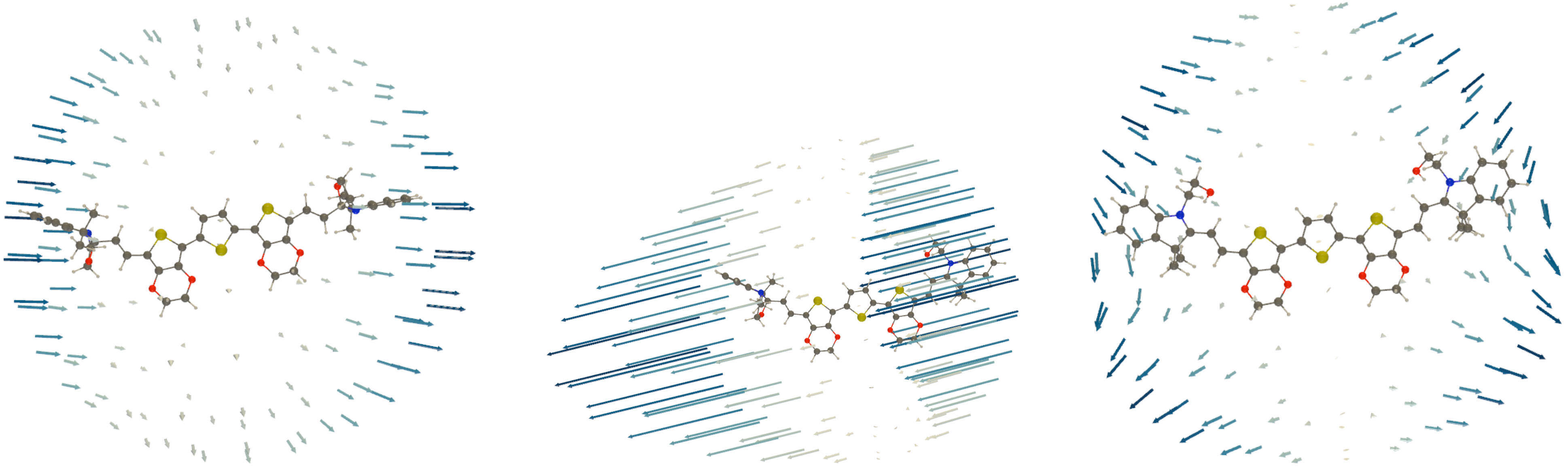

(a) **CC**: $\beta_{HRS} = 1.3$ (6.06), sc=10^{-3}

(b) **CO**: $\beta_{HRS} = 62.0$ (4.96), sc=10^{-4}

(c) **OO**: $\beta_{HRS} = 10.8$ (2.64), sc=10^{-4}

Figure S6: USR (together with β_{HRS} [10^3 a.u., DR in parentheses] and scaling factor [sc, Å a.u.$^{-1}$]) of the dynamic ($\lambda = 1907$ nm) β tensor of the most stable conformers of each form of **2e**, as evaluated at the TDDFT/M06-2X/6-311+G(d)/IEF-PCM (acetonitrile) level of approximation.

	CCC				CCO			
	Static	1907 nm	1300 nm	1064 nm	Static	1907 nm	1300 nm	1064 nm
1	4.1 (1.64)	4.4 (1.58)	5.9 (1.56)	8.5 (1.55)	44.2 (4.50)	53.8 (4.56)	118.3 (4.75)	554.9 (4.97)
2	4.3 (1.87)	4.5 (1.74)	6.0 (1.69)	8.6 (1.65)	43.7 (4.46)	53.4 (4.53)	117.5 (4.74)	548.0 (4.97)
3	4.2 (1.74)	4.5 (1.65)	6.1 (1.63)	8.7 (1.61)	42.4 (4.48)	51.4 (4.53)	112.8 (4.74)	522.8 (4.97)
4	4.2 (1.80)	4.5 (1.69)	6.1 (1.66)	8.6 (1.63)	41.0 (4.43)	50.0 (4.50)	109.2 (4.72)	486.9 (4.96)
5	4.2 (1.81)	4.5 (1.69)	6.1 (1.66)	8.7 (1.63)	42.7 (4.51)	51.2 (4.55)	110.7 (4.75)	487.3 (4.96)
6	4.1 (1.55)	4.5 (1.53)	6.2 (1.53)	8.9 (1.52)	42.8 (4.52)	51.1 (4.55)	111.7 (4.75)	521.7 (4.97)
7	4.1 (1.70)	4.5 (1.62)	6.1 (1.61)	8.7 (1.59)	38.2 (4.41)	46.0 (4.47)	97.1 (4.69)	383.5 (4.93)
8	4.1 (1.61)	4.4 (1.57)	6.0 (1.55)	8.6 (1.54)	39.7 (4.39)	48.8 (4.48)	103.0 (4.70)	400.3 (4.93)
9	4.1 (1.68)	4.5 (1.62)	6.2 (1.61)	8.8 (1.60)	39.7 (4.39)	48.8 (4.48)	103.0 (4.70)	400.4 (4.93)
10	4.1 (1.64)	4.3 (1.59)	5.9 (1.58)	8.4 (1.56)	39.7 (4.40)	47.9 (4.48)	102.1 (4.70)	417.2 (4.94)
11	4.1 (1.65)	4.4 (1.60)	6.0 (1.58)	8.7 (1.57)	39.3 (4.43)	47.3 (4.49)	98.9 (4.70)	375.2 (4.93)
12	4.1 (1.52)	4.5 (1.52)	6.2 (1.52)	8.8 (1.52)	38.8 (4.37)	47.5 (4.47)	101.0 (4.69)	403.3 (4.93)
13	4.1 (1.63)	4.3 (1.58)	5.9 (1.56)	8.4 (1.55)				
14	4.1 (1.60)	4.5 (1.57)	6.1 (1.56)	8.8 (1.55)				
15	4.1 (1.56)	4.5 (1.55)	6.2 (1.54)	8.9 (1.54)				
16	4.1 (1.55)	4.4 (1.54)	6.0 (1.53)	8.6 (1.53)				
17	4.1 (1.59)	4.5 (1.57)	6.1 (1.56)	8.7 (1.56)				
Avg.	4.2 (1.72)	4.4 (1.64)	6.0 (1.61)	8.6 (1.59)	43.1 (4.47)	52.5 (4.54)	114.9 (4.74)	528.1 (4.96)
	COO				OOO			
1	45.1 (2.88)	55.0 (2.88)	116.1 (3.05)	515.6 (2.93)	40.7 (1.56)	51.2 (1.53)	107.5 (1.53)	417.5 (1.54)
2	44.3 (2.96)	53.8 (2.93)	112.8 (3.09)	477.0 (3.02)	38.6 (1.65)	49.1 (1.58)	103.7 (1.56)	405.3 (1.55)
3	45.9 (2.86)	55.9 (2.86)	118.1 (3.02)	537.5 (2.87)	39.9 (1.61)	50.3 (1.55)	105.9 (1.53)	415.0 (1.53)
4	45.2 (2.90)	55.1 (2.88)	116.2 (3.04)	526.1 (2.90)	39.3 (0.00)	49.7 (0.00)	103.3 (0.00)	381.0 (0.00)
5	46.6 (3.07)	56.2 (3.01)	117.8 (3.17)	513.3 (3.02)	39.1 (1.54)	49.5 (1.52)	103.8 (1.52)	399.1 (1.51)
6	47.1 (3.03)	57.1 (2.98)	119.9 (3.14)	538.6 (2.96)	39.0 (1.54)	49.3 (1.51)	103.2 (1.51)	393.5 (1.53)
7					37.5 (1.63)	47.3 (1.57)	98.8 (1.56)	370.3 (1.55)
Avg.	45.4 (2.92)	55.2 (2.91)	116.2 (3.07)	514.4 (2.94)	39.8 (1.52)	50.3 (1.47)	105.7 (1.46)	410.4 (1.46)

Table S19: Computed static and dynamic first hyperpolarizabilities (β_{HRS} in 10^3 a.u., DR in parentheses) for the different conformers of compound **3a**, as evaluated at the TDDFT/M06-2X/6-311+G(d)/IEF-PCM (acetonitrile) level of approximation. The last line contains averaged values using the MB population at 298.15 K as calculated at the ωB97X-D/6-311G(d)/IEF-PCM (acetonitrile) level of theory.

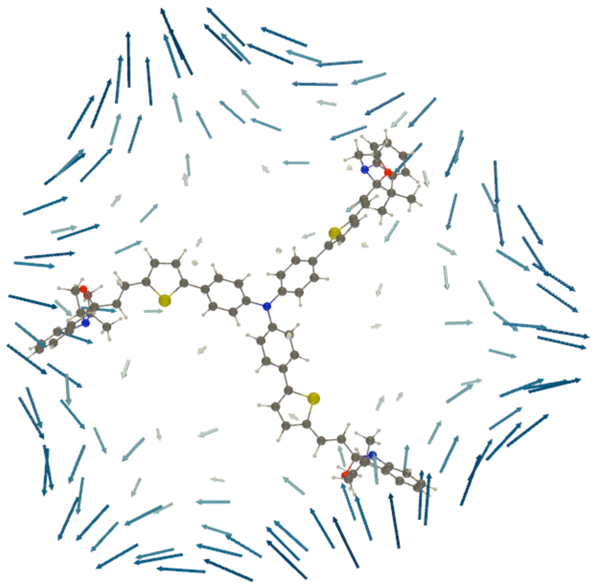

(a) **CCC**: $\beta_{HRS} = 4.4$ (1.58), sc=5×10^{-4}

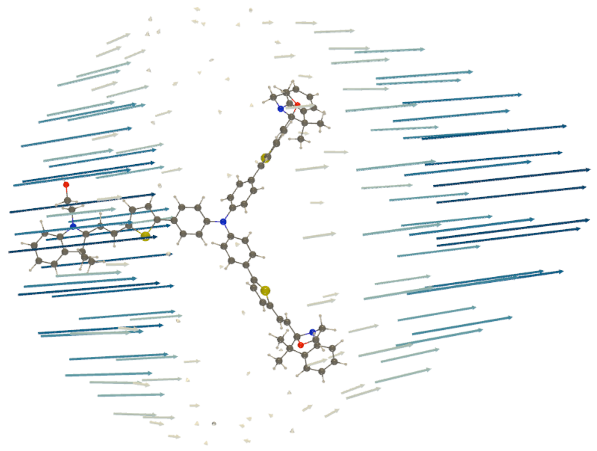

(b) **CCO**: $\beta_{HRS} = 53.8$ (4.56), sc=10^{-4}

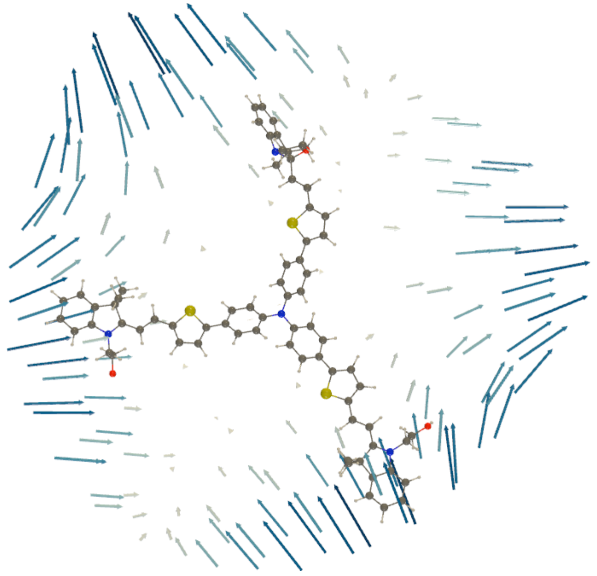

(c) **COO**: $\beta_{HRS} = 55.0$ (2.88), sc=5×10^{-5}

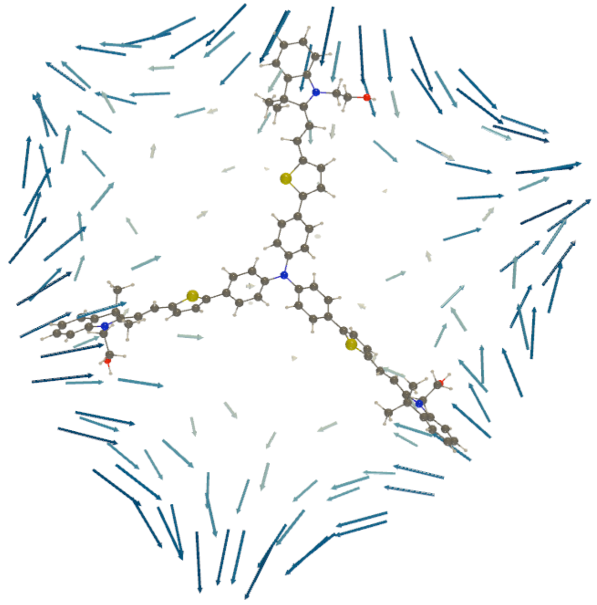

(d) **OOO**: $\beta_{HRS} = 51.2$ (1.53), sc=5×10^{-5}

Figure S7: USR (together with β_{HRS} [10^3 a.u., DR in parentheses] and scaling factor [sc, Å a.u.$^{-1}$]) of the dynamic ($\lambda = 1907$ nm) β tensor of the most stable conformers of each form of **3a**, as evaluated at the TDDFT/M06-2X/6-311+G(d)/IEF-PCM (acetonitrile) level of approximation.

	**CCC** [Ph(C)-BiPh(C)-PhTh(C)]				**CCO** [Ph(C)-BiPh(C)-PhTh(O)]			
	Static	1907 nm	1300 nm	1064 nm	Static	1907 nm	1300 nm	1064 nm
1	3.6 (1.61)	3.7 (1.61)	5.0 (1.62)	6.9 (1.66)	42.0 (4.66)	47.8 (4.70)	106.4 (4.83)	491.8 (4.92)
2	3.6 (1.66)	3.7 (1.63)	5.0 (1.65)	6.9 (1.68)	42.5 (4.66)	48.6 (4.70)	108.9 (4.83)	520.3 (4.93)
3	3.7 (1.67)	3.8 (1.66)	5.1 (1.68)	7.1 (1.71)	42.2 (4.65)	49.6 (4.68)	119.5 (4.85)	512.3 (4.92)
4	3.7 (1.67)	3.9 (1.66)	5.2 (1.68)	7.2 (1.71)	41.7 (4.65)	47.7 (4.69)	105.6 (4.83)	477.9 (4.92)
5	3.6 (1.63)	3.8 (1.62)	5.0 (1.64)	7.0 (1.67)	44.5 (4.58)	51.3 (4.65)	115.2 (4.82)	536.6 (4.99)
6	3.7 (1.66)	3.9 (1.65)	5.2 (1.67)	7.2 (1.70)				
7	3.5 (1.54)	3.7 (1.55)	4.9 (1.56)	6.8 (1.59)				
Avg.	3.6 (1.64)	3.8 (1.63)	5.0 (1.65)	7.0 (1.68)	42.2 (4.66)	48.3 (4.69)	108.8 (4.83)	502.4 (4.92)
	**COC** [Ph(C)-BiPh(O)-PhTh(C)]				**OCC** [Ph(O)-BiPh(C)-PhTh(C)]			
1	28.3 (4.15)	34.4 (4.31)	64.4 (4.53)	154.9 (4.76)	31.7 (4.67)	30.4 (4.72)	56.9 (4.83)	149.8 (4.94)
2	27.4 (4.14)	33.5 (4.32)	62.5 (4.53)	149.1 (4.76)	32.2 (4.65)	31.1 (4.69)	59.0 (4.81)	159.7 (4.93)
3	25.7 (4.06)	31.4 (4.24)	58.0 (4.47)	134.9 (4.71)	32.4 (4.63)	31.5 (4.67)	60.0 (4.80)	164.6 (4.93)
4	27.2 (4.14)	33.3 (4.33)	62.0 (4.54)	147.1 (4.76)	33.2 (4.68)	32.8 (4.74)	62.4 (4.84)	171.2 (4.94)
5	26.4 (4.13)	32.0 (4.30)	59.1 (4.51)	138.3 (4.73)	31.5 (4.65)	30.3 (4.69)	56.6 (4.81)	148.6 (4.93)
Avg.	27.5 (4.13)	33.5 (4.31)	62.5 (4.52)	148.9 (4.75)	32.0 (4.67)	30.8 (4.71)	58.0 (4.82)	154.6 (4.93)
	**COO** [Ph(C)-BiPh(O)-PhTh(O)]				**OCO** [Ph(O)-BiPh(C)-PhTh(O)]			
7	32.2 (2.93)	37.3 (2.91)	78.9 (3.27)	327.6 (3.93)	35.7 (3.24)	36.9 (3.16)	69.7 (3.27)	206.3 (3.11)
8	37.4 (2.96)	43.8 (2.96)	94.2 (3.32)	395.5 (4.11)	35.7 (3.23)	36.9 (3.16)	69.9 (3.27)	207.4 (3.12)
9	32.8 (2.91)	38.2 (2.90)	80.6 (3.25)	333.4 (3.91)	36.2 (3.05)	37.8 (3.02)	71.8 (3.13)	214.4 (2.97)
Avg.	33.8 (2.93)	39.3 (2.92)	83.6 (3.28)	347.8 (3.98)	35.8 (3.23)	36.9 (3.16)	69.8 (3.27)	206.6 (3.11)
	**OOC** [Ph(O)-BiPh(O)-PhTh(C)]				**OOO** [Ph(O)-BiPh(O)-PhTh(O)]			
1	31.3 (3.24)	32.7 (3.16)	57.8 (3.37)	150.1 (3.84)	30.7 (3.24)	32.7 (3.16)	62.2 (3.37)	178.6 (3.84)
2	31.8 (3.31)	32.5 (3.25)	59.7 (3.47)	154.9 (3.93)	31.8 (3.23)	34.4 (3.14)	65.6 (3.38)	189.2 (3.86)
3	31.9 (3.23)	32.4 (3.14)	60.0 (3.38)	159.3 (3.86)				
Avg.	31.4 (3.25)	32.7 (3.16)	58.2 (3.38)	151.3 (3.85)	30.7 (3.24)	32.8 (3.16)	62.4 (3.37)	179.2 (3.84)

Table S20: Computed static and dynamic first hyperpolarizabilities (β_{HRS} in 10^3 a.u., DR in parentheses) for the different conformers of compound **3b**, as evaluated at the TDDFT/M06-2X/6-311+G(d)/IEF-PCM (acetonitrile) level of approximation. The last line contains averaged values using the MB population at 298.15 K as calculated at the ωB97X-D/6-311G(d)/IEF-PCM (acetonitrile) level of theory.

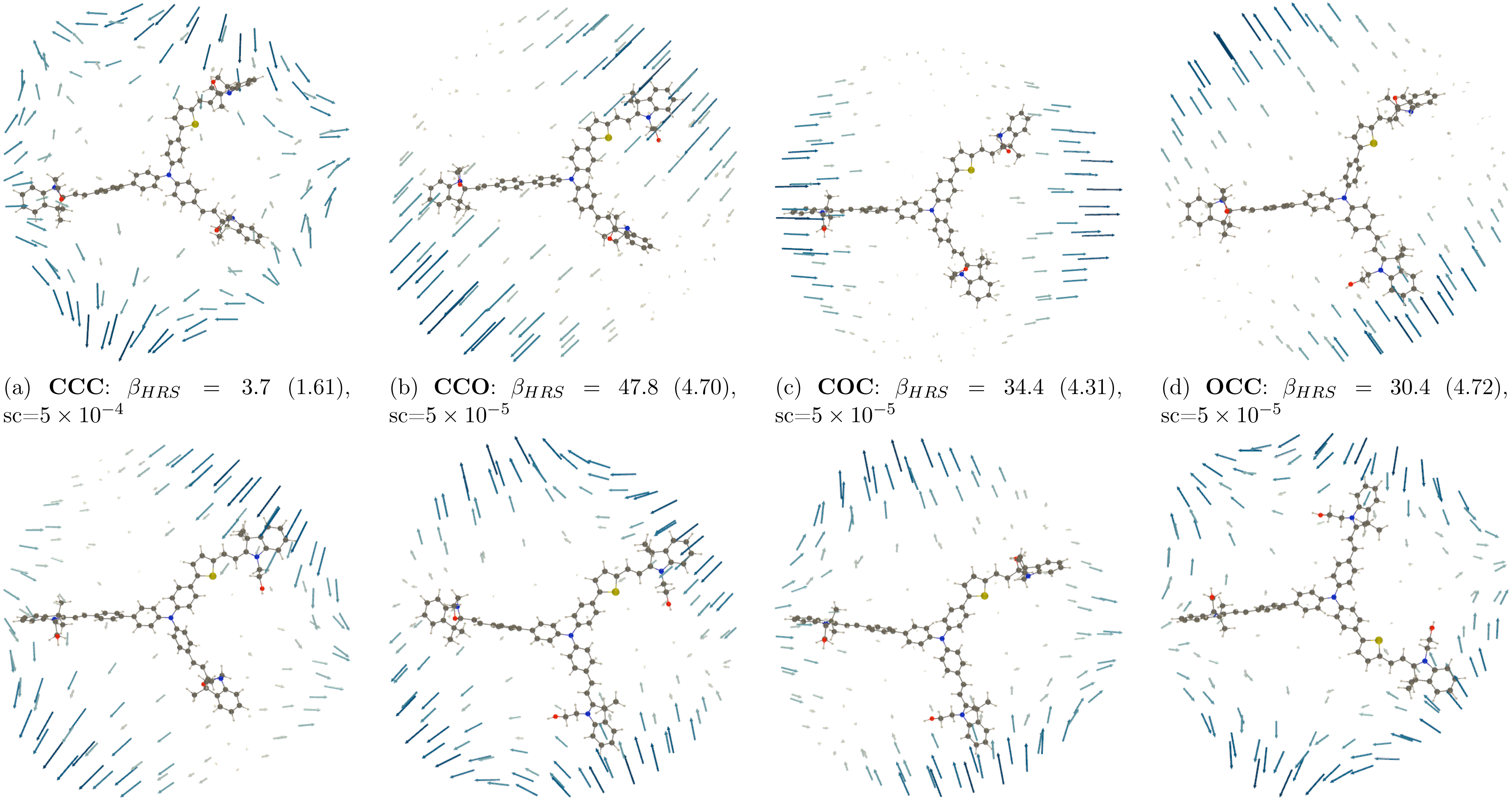

(a) **CCC**: β_{HRS} = 3.7 (1.61), sc=5×10^{-4}

(b) **CCO**: β_{HRS} = 47.8 (4.70), sc=5×10^{-5}

(c) **COC**: β_{HRS} = 34.4 (4.31), sc=5×10^{-5}

(d) **OCC**: β_{HRS} = 30.4 (4.72), sc=5×10^{-5}

(e) **COO**: β_{HRS} = 37.2 (2.91), sc=5×10^{-5}

(f) **OCO**: β_{HRS} = 36.9 (3.16), sc=5×10^{-5}

(g) **OCC**: β_{HRS} = 32.7 (3.16), sc=5×10^{-5}

(h) **OOO**: β_{HRS} = 32.7 (3.16), sc=5×10^{-5}

Figure S8: USR (together with β_{HRS} [10^3 a.u., DR in parentheses] and scaling factor [sc, Å a.u.$^{-1}$]) of the dynamic ($\lambda = 1907$ nm) β tensor of the most stable conformers of each form of **3b**, as evaluated at the TDDFT/M06-2X/6-311+G(d)/IEF-PCM (acetonitrile) level of approximation.

S3 Excitation energies

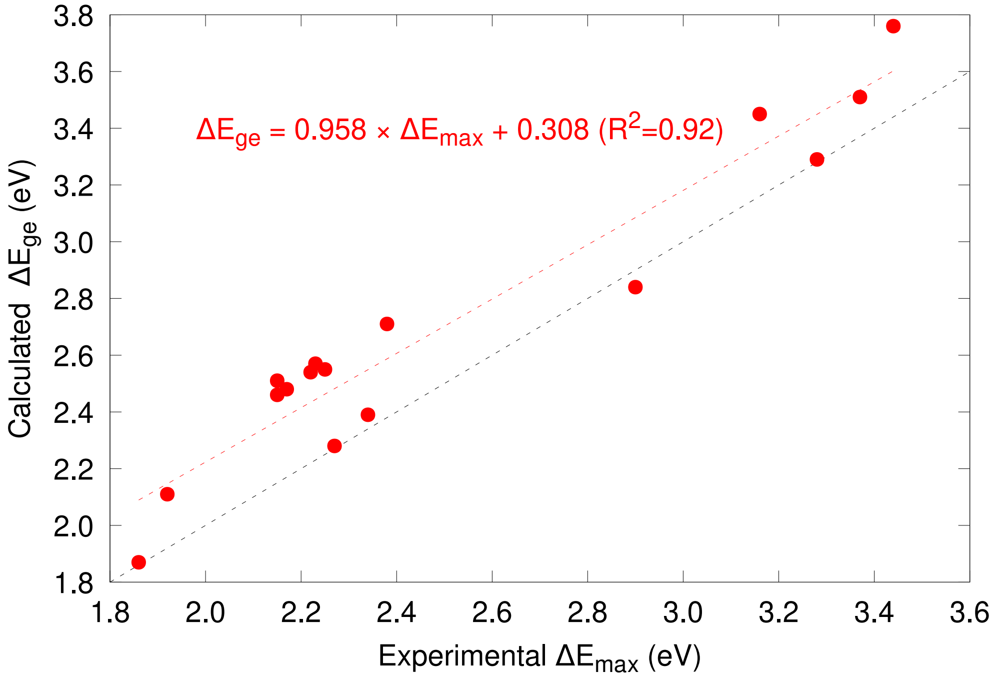

Figure S9: Correlation (in dashed red) between experimental first maximum absorption energies (ΔE_{max}, eV) and calculated first vertical transition energies (ΔE_{ge}), as evaluated at the TDDFT/M06-2X/6-311+G(d)/IEF-PCM (acetonitrile) level of approximation. This includes compounds **1** (experimental values from Ref. [1], for R=Me, calculated values for R=H recomputed from our conformers), **2a** and **2e** (experimental and calculated values from Ref. [2]), and **3a-3b** (experimental and calculated values from Ref. [3]). The dashed black line represents perfect agreement.

References

[1] Pielak, K. *et al.* Second-Order Nonlinear Optical Properties of Multiaddressable Indolinooxazolidine Derivatives: Joint Computational and Hyper-Rayleigh Scattering Investigations. *J. Phys. Chem. C* **121**, 1851–1860 (2017).

[2] Quertinmont, J. *et al.* Combining Benzazolo-Oxazolidine Twins toward Multi-state Nonlinear Optical Switches. *J. Phys. Chem. B* **125**, 3918–3931 (2021).

[3] Aidibi, Y. *et al.* A molecular loaded dice: When the π conjugation breaks the statistical addressability of an octastate multimodal molecular switch. *Dyes Pigments* **202**, 110270 (2022).
